# Metastasis to the stomach: a systematic review

**DOI:** 10.12688/f1000research.140758.1

**Published:** 2023-10-18

**Authors:** Arturan Ibrahimli, Altay Aliyev, Aykhan Majidli, Aysegul Kahraman, Aysuna Galandarova, Emil Khalilzade, Heydar Mammadli, Kamran Huseynli, Karam Assaf, Cagatay Kilinc, Nijat Muradov, Omer F. Alisan, Sabir Abdullayev, Yeliz I. Sahin, Elgun Samadov

**Affiliations:** 1 Liv Bona Dea Hospital, Baku, Azerbaijan

**Keywords:** Metastasis to stomach, gastric metastases, gastric metastasis, gastric cancer, stomach cancer

## Abstract

**Background:** This study reviews the literature on gastric metastases (GM) in terms of diagnosis, treatment, and outcomes. The goal of this study was to provide clinicians with a reliable and beneficial source to understand gastric metastases arising from various primary tumors and to present the growing literature in an easily accessible form.

**Methods:** Articles published in English language from implementation of MEDLINE and Cochrane databases until May 2022 were considered for the systematic review. Articles other than English language, letters to the editor, posters, and clinical images were excluded. Hematogenous and lymphogenic metastases were included whereas direct tumoral invasion and seeding were excluded. Articles and abstracts were analyzed and last selection was done after cross-referencing and by use of defined eligibility criteria.

**Results:** In total 1,521 publications were identified and 170 articles were finally included totaling 186 patients with GM. The median age of patients was 62 years. Gynecologic cancer was the most common cancer type causing GM (67 patients), followed by lung cancer (33 patients), renal cancer (20 patients), and melanoma (19 patients). One of the main treatment methods performed for metastasis was resection surgery (n=62), sometimes combined with chemotherapy (ChT) or immunotherapy. ChT was the other most used treatment method (n=78). Also, immunotherapy was amongst the most preferred treatment options after surgery and ChT (n=10).

**Conclusions:** As 172 case reports were screened in the systematic review from different journals, heterogeneity was inevitable. Some articles missed important information such as complete follow-up or clinical information. Moreover, since all of the included articles were case reports quality assessment could not be performed. Among 172 case reports reviewed, resection surgery was performed the most and was sometimes combined with ChT and immunotherapy. Further research about what type of treatment has the best outcomes for patients with gastric metastases is needed.

## Introduction

Metastases to the stomach are rare conditions with poor prognosis that may present with both gastrointestinal and systemic symptoms, such as loss of appetite, abdominal pain, fatigue, nausea, and vomiting, with a reported incidence of 0.2-0.7% based on clinical and autopsy findings. ^1^ Gastrectomy is thought to be the only potentially curative treatment for metastatic gastric cancer but the primary site of the tumor is also considered along with the type and grade of the tumor when planning treatment in gastric metastases. Therefore, chemotherapy is also an option for patients with higher grades and multi-focal cancers. This study reviews the literature on gastric metastases in terms of diagnosis, treatment, and outcomes. The intended goal of this study was to provide clinicians with a reliable and beneficial source to understand gastric metastases arising from various primary tumors and to present the growing literature in an easily accessible form by reviewing the case reports of different primary tumors separately with consideration of diagnosis, treatment, and clinical presentation which may vary from patient to patient depending on primary site of the tumor.

## Methods

This systematic review adheres to the Preferred Reporting Items for Systematic Reviews and Meta-Analyses (PRISMA) guidelines. ^172^ A computerized literature search through MEDLINE/ PubMed and Cochrane databases was conducted until May 2022.

The following combination of keywords was used for the search: ({({gastric (MeSH Terms)} AND {neoplasm metastasis (MeSH Terms)}) OR (gastric metastasis)} OR {gastric metastases}) OR (metastasis to the stomach). The search was limited by filtering for “free full text” and “case reports.” After the decision of inclusion and exclusion criteria by the team, two of the reviewers independently screened and retrieved each report.

Hematogenous and lymphogenic metastases were included whereas direct tumoral invasion and seeding were excluded from the study. Articles other than the English language, letters to the editor, posters, and clinical images were excluded. After the studies were screened and separated based on the inclusion and exclusion criteria, reviewers were divided to groups based on primary tumor location. Each group contained two reviewers to collect the data from studies of its specific location for example metastasis from gynecologic cancers or lung cancers.

The following data were extracted from the databases: first author, number of cases, age, sex, site of the primary tumor, histology and treatment of primary tumor, treatment of metastasis, clinical presentation of gastric metastases (GM), synchronous or metachronous GM, the time between primary and secondary GM, diagnostic procedures, other metastasis, and overall survival.

Since the study only contains screening of case reports, assessment of bias risk was not performed and thus it is mentioned as a limitation of study in discussion section.

## Results

**Figure 1.**
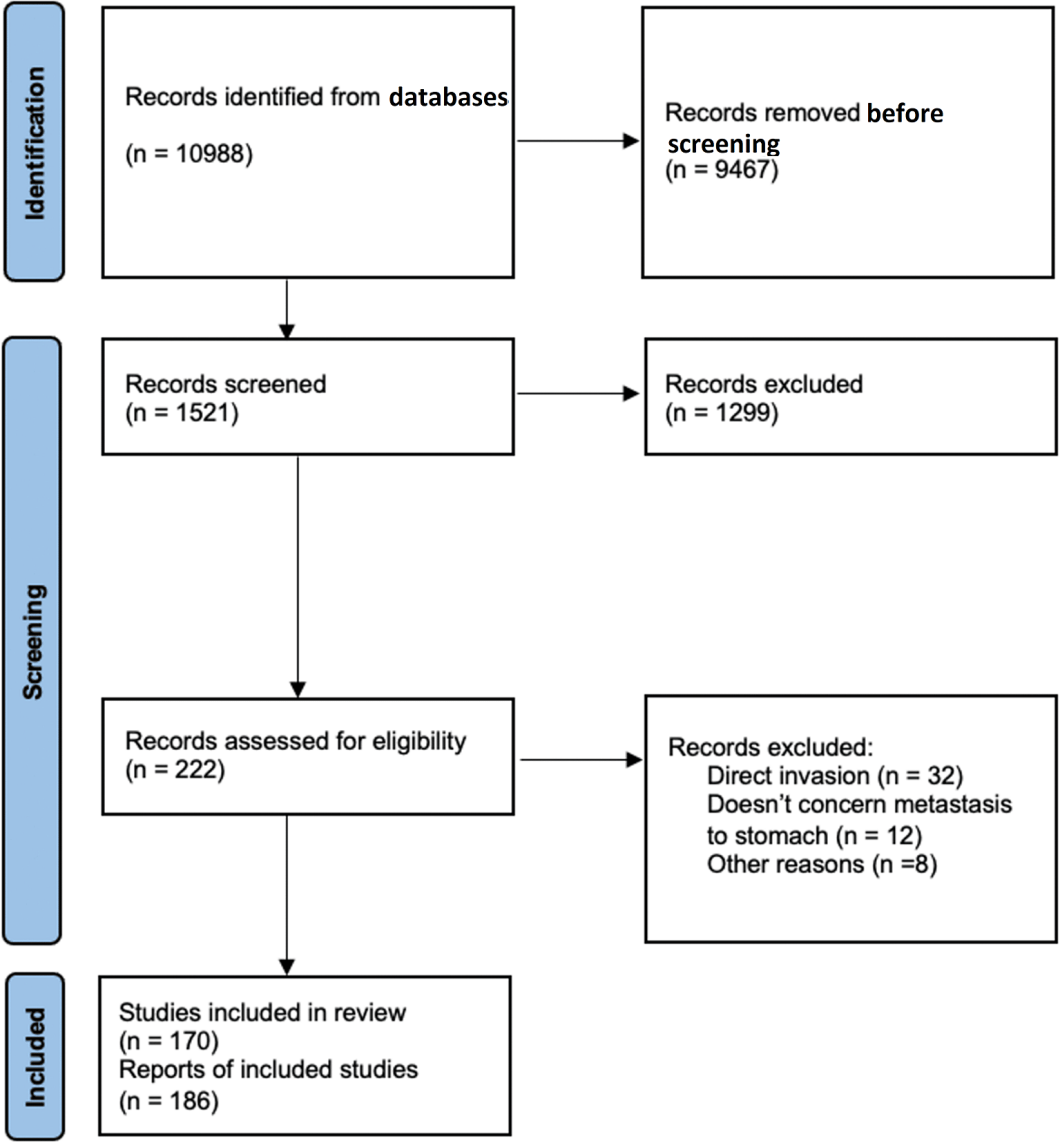
Preferred Reporting Items for Systematic Reviews and Meta-Analyses (PRISMA) flow chart.

The PRISMA flow chart below illustrates details about data collection ( Figure 1).

**Figure 2.**
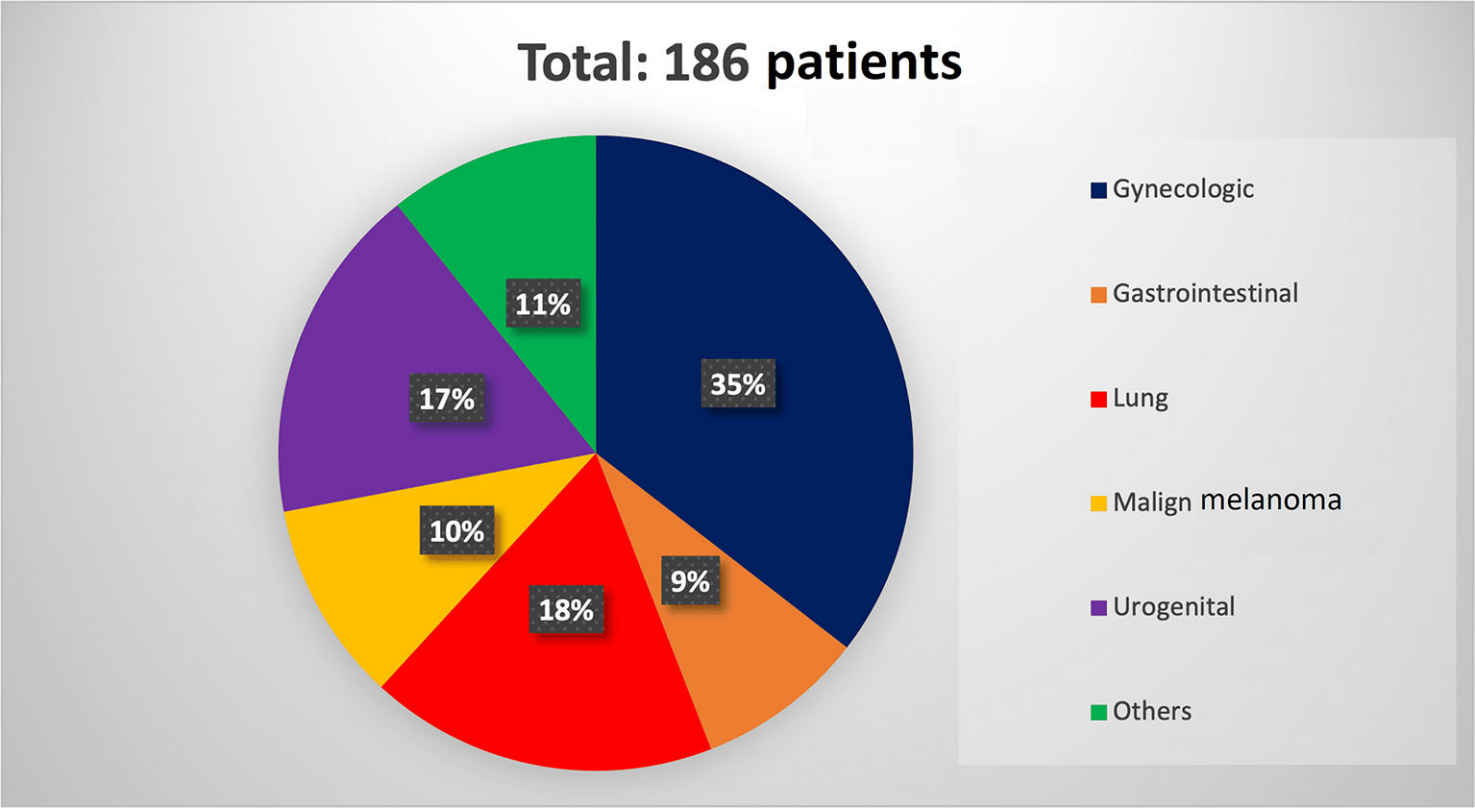
Pie chart demonstrating percentages of primary tumor sites of patients with metastasis to stomach.

In total, 1,521 publications were identified and 170 articles were finally included totaling 186 patients with GM (101 female and 85 male). The median age of patients was 62 years (IQR: 55-70.5). Gynecologic cancer (including breast cancer) was the most common cancer type causing GM (66 patients), followed by lung cancer (33 patients), renal cancer (20 patients), and melanoma (19 patients) ( Figure 2). Results are presented below according to the origin of the primary tumor. The main treatment method performed for metastasis was resection surgery (n=62, total, subtotal or partial gastrectomy, proximal gastrectomy, radical total gastrectomy with Roux-en-Y, wedge gastrectomy, and laparoscopic resection of gastric metastasis), sometimes combined with chemotherapy (ChT) or immunotherapy. Chemotherapy was the other most used treatment method (n=78). Also, immunotherapy was among the most preferred treatment options after surgery and chemotherapy (n=10).

### Gynecologic cancer

The median age of the 66 patients was 57 years. In total, 46 cases had metastases other than GM. Bone was the most common site of metastasis. Five cases had no other metastases. The total number of cases in the breast group was 54, and one of them was a male patient. The median age of the breast group is 56, the youngest patient was 36 years old and the oldest patient was 84 years old. Invasive lobular carcinoma (ILC) had the largest number of patients in comparison to ovarian and uterine groups. A total of 31 patients presented with ILC. The ovarian group had nine patients; the median age was 61 years. The oldest patient was 73 years old; the youngest patient was 47 years old. The uterine group had two patients with ages 49 and 80 years. In most cases, systemic therapy was more effective than surgery. Surgical treatment had a role in palliative treatment. As a systemic treatment, chemotherapy was the most utilized treatment. Overall survival was given in only 25 cases and ranged from a few days to nine years. Six of the total patients are still alive. Table 1 ^2^^–^^56^ summarizes the findings of included studies regarding gynecologic cancers.

**Table 1.** Illustrating the data regarding metastasis from gynecologic cancers.

First author	No of cases	Age	Sex	Site of primary tumor	Histology type of primary	Treatment of primary	Treatment of metastasis	Clinical presentation of GM
Fousekis et al. ^2^	1	64	F	Breast	Lobular Ca	ChT	ChT	Dysphagia, dyspepsia
Watanabe et al. ^3^	1	71	F	Breast	Ductal Ca	Mastectomy and axillary lymph adenectomy, ChT	Endocrine therapy	Asymptomatic
Husain et al. ^4^	1	47	F	Left breast	Ductal Ca	Neoadjuvant ChT, mastectomy with a left axillary lymph adenectomy, adjuvant endocrine therapy	N/S	Dyspepsia, weight loss, vomiting
Zhang et al. ^5^	1	46	F	Bilateral breast	Lobular Ca	N/A	N/A	Epigastric discomfort
Jabi et al. ^6^	1	60	F	Right breast	Lobular Ca	Palliative ChT	Palliative ChT	Epigastralgia, gastric bleeding, Anemia
Johnson et al. ^7^	1	50	F	Breast	Ductal Ca	Lumpectomy, adjuvant RT, ChT	N/S	N/S
Okamoto et al. ^8^	1	51	F	Breast	Ductal Ca	ChT	ChT	Melena, presyncope
Nehmeh et al. ^9^	1	58	F	Right breast	Ductal Ca	Right modified mastectomy, left prophylactic mastectomy, adjuvant ChT	N/S	Perforated ulcer
Hanafiah et al. ^10^	1	71	F	Left breast	Lobular Ca	Left mastectomy, axillary clearance, ChT, RT	Ch	Hoarseness, weight loss, early satiety
Teixeira et al. ^11^	1	40	F	Right breast	Lobular Ca	Neoadjuvant ChT, RT, conservative surgery for right breast and right axillary lymph node	Total gastrectomy	Nausea, epigastric discomfort, early satiety, weight loss
Kutasovic et al. ^12^	1	52	F	Left breast	Invasive Ca of no special type	Local excision, adjuvant RT, ChT, hormone therapy	Subtotal gastrectomy	N/S
Abdallah et al. ^13^	1	53	F	Breast	Lobular Ca	ChT, hormone therapy	N/S	Abdominal pain, diffuse tenderness, abdominal distention
Liu et al. ^14^	1	82	F	Left breast	Phyllodes tumors	Total mastectomy for recurrent tumor local excision, RT	Excision surgery, RT	Anemia, melena
Tang et al. ^15^	1	67	F	Left breast	Ductal Ca	Left breast-conserving surgery, axillary lymphadenectomy, adjuvant ChT, RT	N/S	Stomach pain
De Gruttola et al. ^16^	1	61	F	Breast	Lobular Ca	Mastectomy, adjuvant ChT, RT	Total gastrectomy due to gastric perforation	Gastric perforation
Mohy-Ud-Din et al. ^17^	1	83	F	Breast	Lobular Ca	Mastectomy, sentinel lymph nodes excision, adj. ChT	N/A	Nausea, vomiting
Güler et al. ^18^	1	42	F	Breast	Ductal Ca	N/A	Total gastrectomy due to gastric perforation, ChT	Acute Abdomen
Cui et al. ^19^	1	42	F	Endometrium	Endometrial Aca	Total hysterectomy, bilateral salpingo-oophorectomy, pelvic and para-aortic lymphadenectomy	Neoadjuvant ChT, partial gastrectomy	N/S
Asmar et al. ^20^	1	84	F	Breast	Lobular Ca	Left mastectomy, adjuvant ChT, RT, hormone therapy	Hormone therapy	Dyspepsia
Klair et al. ^21^	1	60	F	Ovary	Ovarian granulosa cell tumor	Total hysterectomy, bilateral salpingo-oophorectomy	N/A	Reflux, abdominal pain, nausea, anorexia
Yang ^22^	1	47	F	Ovary	Ovarian serous cystadenocarcinoma	Total hysterectomy, bilateral salpingo-oophorectomy, pelvic and paraaortic lymphadenectomy, total omentectomy	Laparoscopic resection, adjuvant ChT	Abdominal pain
Bushan et al. ^23^	1	68	F	Left breast	Lobular Ca	Wide excision of breast lesion, ChT, RT, hormone therapy	Distal gastrectomy with D2 lymphadenectomy, left axillary excision, ChT	Weight loss, dysphagia
Zhang et al. ^24^	2	45	F	Breast	Lobular Aca	Lumpectomy, RT, ChT	N/S	N/S
		64	F	Breast	Lobular Aca	N/A	N/A	Weight loss
Jin et al. ^25^	1	55	F	Breast	Lobular Ca	Neoadjuvant ChT, radical mastectomy, ChT, RT	ChT	N/S
Buka et al. ^26^	1	58	F	Breast	Invasive lobular Ca	ChT, hormone therapy, RT	Neoadjuvant ChRT, total gastrectomy, adjuvant ChT	Abdominal pain, weight loss
Dória et al. ^27^	1	66	F	Breast	Invasive lobular Ca	Letrozole	Total gastrectomy, lymphadenectomy, esophagojejunostomy with a Roux loop technique	Epigastric pain, vomiting, weight loss
Hwangbo et al. ^28^	1	73	F	Ovary	Serous Aca	Cytoreductive surgery, adjuvant ChT	Distal gastrectomy with Billroth I anastomosis, lymphadenectomy	Epigastric pain, dyspepsia
Shetty et al. ^29^	2	56	F	Breast	Invasive ductal Ca	Breast conservation therapy, adjuvant ChT	ChT	Epigastric discomfort, non-bilious vomiting
		61	F	Breast	Invasive ductal Ca	Left breast modified radical mastectomy, adjuvant RT	Palliative ChT	Abdominal pain, melena, abdominal distension
Geredeli et al. ^30^	1	47	F	Breast	Invasive lobular Ca	Palliative ChT	Subtotal stomach resection, ChT	Asymptomatic
Kim et al. ^31^	1	58	F	Ovary	Serous Aca	Total hysterectomy with salpingo-oophorectomy, lymphadenectomy with total omentectomy, adjuvant ChT	Subtotal gastrectomy, lymphadenectomy, ChT	Asymptomatic
Fernandes et al. ^32^	1	51	F	Breast	Invasive lobular Ca	Quadrantectomy, adjuvant ChT, adjuvant RT, hormone therapy	Total gastrectomy, adjuvant ChT, hormone therapy	Dyspepsia
Moldovan et al. ^33^	1	49	F	Cervix uteri	SCC	Surgery, ChRT	Subtotal gastrectomy, lymphadenectomy D2, anastomotic layout shaped as Y Roux, omentectomy, adjuvant ChRT	Pyloric stenosis, epigastric pains, late postprandial emesis, weight loss
Zhou and Miao ^34^	1	61	F	Ovary	Serous Aca	Optimal debulking cytoreductive surgery, adjuvant ChT	Gastric antrectomy	Asymptomatic
Critchley et al. ^35^	1	62	F	Breast	Invasive lobular Ca	Mastectomy, level 2 axillary clearance, adjuvant ChT, adjuvant RT, adjuvant hormone therapy	ChT	Loose stool, normocytic anemia, weight loss
Hara et al. ^36^	1	74	F	Breast	Invasive ductal Ca	Breast-conserving surgery	Paclitaxel	Chronic gastritis
Ciulla et al. ^37^	1	70	F	Breast	Lobular Ca	Postoperative hormone therapy	Total gastrectomy, lymphadenectomy R1, esophagojejunostomy with Roux loose technique	Asymptomatic
Jones et al. ^38^	2	51	F	Breast	Lobular Ca	Wide local excision, axillary dissection, adjuvant RT	Total gastrectomy with Roux-en-Y reconstruction, hormone therapy	Weight loss, epigastric pain
		61	F	Breast	Lobular Ca	Mastectomy, axillary dissection, adjuvant ChT, RT, tamoxifen	ChT, RT	Progressive dysphagia, weight loss
Yim et al. ^39^	1	48	F	Breast	SRCC	ChT	ChT	Epigastric discomfort
Wong et al. ^40^	1	72	F	Breast	Invasive lobular Ca	Wide local excision, adjuvant RT	Hormone therapy	Acute abdomen, rebound tenderness, generalized peritonitis
Ricciuti et al. ^41^	1	65	M	Breast	Invasive ductal Ca	Total mastectomy, complete axillary dissection, adjuvant hormone therapy	Gastrectomy with Roux-en-Y esophagojejunostomy anastomosis	Hematemesis, epigastric pain
Fernandes et al. ^42^	4	56 (the mean age)	F	Breast	Invasive lobular Ca	ChT, hormone therapy	Total gastrectomy	Ulcerated lesion, major bleeding
		56 (the mean age)	F	Breast	Invasive lobular Ca	ChT, RT, hormone therapy	ChRT	Diffuse infiltration
		56 (the mean age)	F	Breast	Invasive ductal Ca	ChT, hormone therapy	ChT	Infiltrative, ulcerated, stenotic lesion
		56 (the mean age)	F	Breast	Invasive ductal Ca	ChT, hormone therapy	ChRT	Flat erosive lesion
Zullo et al. ^43^	3	49	F	Ovary	Serous Aca	Hysterectomy, bilateral salpingo-oophorectomy, pelvic lymphadenectomy, adjuvant ChT	ChT	Abdominal pain, vomiting, weight loss
		80	F	Cervix uteri	Leiomyosarcoma	Total hysterectomy, bilateral salpingo-oophorectomy, pelvic lymphadenectomy, adjuvant ChT	N/A	Epigastric pain
		70	F	Breast	N/A	Radical left mastectomy, adjuvant ChT	ChT	Dysphagia, epigastric pain
Villa Guzman et al. ^44^	1	58	F	Breast	Invasive lobular Ca	Quadrantectomy, lymphadenectomy, adjuvant ChT, RT	ChT, hormone therapy	Nausea, epigastric pain
Mizuguchi et al. ^45^	1	71	F	Ovary	Serous Aca	Total hysterectomy, bilateral salpingo-oophorectomy, omentectomy, ChT	ChT, hormone therapy	Asymptomatic
Jmour et al. ^46^	4	51	F	Breast	Mixed	Radical mastectomy with lymphadenectomy	ChT, RT	Nausea, vomiting, abdominal pain
		47	F	Breast	Lobular infiltrating Ca	Radical mastectomy with lymphadenectomy	ChT, RT	Nausea, vomiting, abdominal pain
		51	F	Breast	Ductal infiltrating Ca	N/A	ChT, RT	Nausea, vomiting, abdominal pain
		36	F	Breast	Lobular infiltrating Ca	Radical mastectomy with lymphadenectomy	ChT, RT	Nausea, vomiting, abdominal pain
Yim ^47^	1	65	F	Breast	Invasive lobular Ca	Modified radical mastectomy, adjuvant ChT, adjuvant RT	ChT	Dyspepsia, anorexia, indigestion, epigastric discomfort, early satiety, weight loss
Choi et al. ^48^	1	44	F	Breast	Phyllodes tumor	Right lumpectomy, axillary lymphadenectomy, RT, right total mastectomy	Endoscopic hemostasis with cauterization	Dizziness, anemia, melena
Khan et al. ^49^	1	56	F	Breast	Signet ring Aca	ChT	ChT	Anemia
Mullally et al. ^50^	1	46	F	Breast	Invasive ductal Ca	Left mastectomy, adjuvant ChT, hormone therapy, RT	Palliative laparoscopic gastroduodenostomy, hormone therapy, palliative ChT	Epigastric and left shoulder pain, epigastric tenderness, upper abdominal rigidity
Kliiger and Gorbaty ^51^	1	60	F	Breast	Invasive ductal Aca	Systemic therapy, ChT	N/A	Nausea, diarrhea, vomiting, weight loss
Antonini et al. ^52^	1	61	F	Ovary	Serous Ca	ChT, cytoreductive surgery	ChT	Dyspepsia
Kono et al. ^53^	1	64	F	Ovary	Mucinous Ca	Bilateral salpingo-oophorectomy, simple hysterectomy, pelvic and para-aortic lymphadenectomy, partial omental resection	ChT	Back pain
Kim et al. ^54^	1	39	F	Breast	Invasive lobular Ca	Right breast-conserving surgery, lymphadenectomy	Duodenal stent, systemic ChT	Upper abdominal discomfort and pain, indigestion
Woo et al. ^55^	1	51	F	Breast	Invasive lobular Ca	Bilateral modified radical mastectomy, ChT, RT	Radical subtotal gastrectomy with Billroth II anastomosis, D2 lymphadenectomy, ChT	Epigastric pain
Ulmer et al. ^56^	1	55	F	Breast	Invasive lobular Ca	Bilateral mastectomy, adjuvant ChT, RT, hormone therapy	Palliative pyloric stent	Nausea, vomiting, early satiety, weight loss

Aca: adenocarcinoma; Ca: carcinoma; SCC: squamous cell carcinoma; GM: gastric metastasis; RT: radiotherapy; ChT: chemotherapy; SRCC: signet ring cell adenocarcinomas; ER: estrogen receptor; HER: human epidermal growth factor receptor; PGR: progesterone receptor; Syn: synchronous; Met: metachronous; EGD: esophagogastroduodenoscopy; FNA: fine-needle aspiration.

### Gastrointestinal cancer

Median age of the 16 patients (11 male, five female) was 69 years, ranging from 22 years to 85 years. Overall survival of the seven patients whose data were given ranged from two months to 16 months. Although, there were six cases who were still alive and the survival of three cases was not reported. Among histological types of gastrointestinal cancers, adenocarcinoma (Adeno Ca) was the most common cancer type (seven patients), followed by hepatocellular cancer (HCC) (four patients) and squamous cell carcinoma (two patients). Endoscopy is the most frequently used method in the diagnosis of metastases. Methods such as computer tomography (CT), positron emission tomography and computed tomography (PET-CT), and endoscopic ultrasound were also used for diagnosis. One patient underwent laparotomy and biopsy. According to this research nine of these patients had surgery. Transcatheter left gastric artery embolization was performed in one patient. On the other hand, seven patients received chemotherapy and one patient had palliative radiotherapy. Nevertheless, one patient is unknown. Findings regarding gastrointestinal cancers are summarized in Table 2. ^57^^–^^72^

**Table 2.** Illustrating data regarding metastasis from gastrointestinal cancers.

First author	No of cases	Age	Sex	Site of primary tumor	Histology type of primary	Treatment of primary	Treatment of metastasis	Clinical presentation of GM
Iwai et al. ^57^	1	76	F	Transverse colon	Poorly differentiated Aca with a partial component of signet-ring Ca	ChT	ChT	Anemia, anorexia
Yang et al. ^58^	1	74	F	Head of pancreas	Poorly differentiated invasive Aca	ChT	ChT	RUQ pain
Lee and Lee ^59^	1	82	M	Right colon	Moderately differentiated Aca	Extended right hemicolectomy (declined adjuvant ChT)	Radical total gastrectomy (declined adjuvant ChT) with Roux-en-Y and D2 dissection	Asymptomatic
Rothermel et al. ^60^	1	61	M	Body of pancreas	Well-differentiated ductal Aca	Distal pancreatectomy, splenectomy, adjuvant ChT	ChT, palliative radiation, and wedge gastrectomy	Asymptomatic
Terashima et al. ^61^	1	61	F	Transverse colon	Poorly differentiated Aca	Extended right hemicolectomy, ChT	Partial gastrectomy and D3 dissection, ChT	Diarrhea, vomiting
Sasajima et al. ^62^	1	72	M	Head and tail of pancreas	IPMN	ChT	ChT (terminated after 2 courses)	N/A
Tomonari et al. ^63^	1	78	M	Body and distal pancreas	Moderately differentiated ACa T3N0M0	Surgery, adjuvant ChT	Subtotal gastrectomy	Follow-up
Adachi ^64^	1	67	F	Pancreas	Well-differentiated SCC	Distal pancreatectomy and splenectomy	Total gastrectomy	Anorexia, back pain
Nakazawa et al. ^65^	1	59	M	Esophagus	Mucosal SCC	Subtotal esophagectomy, left lateral segmentectomy of liver, pancreatosplenectomy, adjuvant ChT	Proximal gastrectomy	Asymptomatic
Abouzied et al. ^66^	1	69	M	Liver	HCC	Right hepatectomy	ChT	Iron-deficiency anemia
Ito et al. ^67^	1	78	M	Liver	ICC	Lateral hepatectomy	Proximal gastrectomy and lymphadenectomy	Fatigue
Imai et al. ^68^	1	62	M	Liver	HCC	N/A	Transcatheter left gastric artery embolization	Abdominal mass
Kim et al. ^69^	1	75	M	Liver	HCC	Right hemihepatectomy, TACE	Gastric wedge resection	Melena, mild dyspnea
Peng et al. ^70^	1	22	M	Liver	HCC	Right hemihepatectomy combined with left lateral tumor local resection, cholecystectomy, splenectomy	Gastric tumor local resection	Anemia, FOBT 4+
Kanthan et al. ^71^	1	85	M	Colon	Aca	N/A	N/A	Anemia
Wang et al. ^72^	1	63	F	Gallbladder	Melanoma	Surgery, ChT	ChT	Postprandial nausea, vomiting

Aca: adenocarcinoma; IPMN: intraductal papillary mucinous neoplasm; HCC: hepatocellular carcinoma; ChT: chemotherapy; RUQ pain: right upper quadrant pain; Syn: synchronous; EUS: endoscopic ultrasound; EUS-FNA: endoscopic ultrasound fine-needle aspiration; Ca: carcinoma; SCC: squamous cell carcinoma; ICC: intrahepatic cholangiocarcinoma; TACE: trans arterial chemoembolization; FOBT: fecal occult blood test; Met: metachronous; EGD: esophagogastroduodenoscopy; FDG: fluorodeoxyglucose; PET: positron emission tomography.

### Lung cancer

The median age of the 33 patients (25 male, eight female) was 62, ranging from 39 years to 78 years. Twenty- seven of the total cases had other metastases in addition to gastric ones. The survival time of the 22 patients whose data were given ranged from two weeks to 30 months. Yet, there were two cases that were still alive four and five years after metastases were found, respectively. Among histological types of primary lung cancers that lead to gastric metastases, adenocarcinoma was the most typical diagnosis (13 patients), followed by small cell lung cancer (SCLC) and squamous cell carcinoma (SCC). Regarding the treatment of GM, different combinations of chemotherapy were the most common choice (15 patients). On the other hand, seven of the total cases received surgical treatment (one esophagogastrostomy, two total, and four partial gastrectomies). However, since one patient’s metastasis was diagnosed after an autopsy, he could not receive any gastric treatment. Moreover, one patient refused any metastasis treatment, while six other cases’ treatments are unknown. Data pertaining to GM originating from primary lung cancers are summarized in Table 3. ^73^^–^^102^

**Table 3.** Illustrating data regarding metastasis from lung cancers.

First author	No of cases	Age	Sex	Site of primary tumor	Histology type of primary	Treatment of primary	Treatment of metastasis	Clinical presentation of GM
Catalano et al. ^73^	1	78	M	Lung - right upper lobe	Poorly differentiated Aca	Upper right lobectomy	Total gastrectomy	Asymptomatic
Shih-Chun et al. ^74^	1	55	M	Lung - right upper lobe	NSCLC	Concurrent chemoradiotherapy	Palliative total gastrectomy, ChT	Gastric bleeding, ulcerative mass
Das Majumdar et al. ^75^	1	72	M	Lung	Poorly differentiated Aca	Palliative RT	Immunotherapy, ChT	Identified with body CT after pathological fracture
Liu et al. ^76^	1	58	M	Lung	Aca	Middle right lobectomy, neoadjuvant therapy, ChT	ChT, partial gastrectomy	N/S
Nemoto et al. ^77^	1	64	M	Lung - right lower lobe	SCC	Adjuvant ChT	Esophagogastrostomy	Epigastric pain, progressive dysphagia
He et al. ^78^	1	61	M	Lung	SCLC	Left lower lobectomy	Cardia resection	Progressive dysphagia
Yang et al. ^79^	1	59	M	Lung - left upper lobe	Poorly differentiated metastatic carcinoma	ChT	Anti-PD1 immunotherapy	Right upper limb pain, epigastric discomfort
Li et al. ^80^	1	61	M	Lung - right lower lobe	SCC	ChT	ChT, gastrectomy	Progressive abdominal distention
Bhardwaj et al. ^81^	1	39	F	Lung	SCC	ChT, nivolumab	RT	Dizziness, melena
Badipatla et al. ^82^	1	65	M	Lung	Aca	ChT, palliative care	ChT, palliative care	Bilateral flank pain, nausea, vomiting, change in bowel habit
Qasrawi et al. ^83^	1	69	F	Lung - left upper lobe	Aca	RT	Hospice care	Melena, hypotension
Kim et al. ^84^	1	70	F	Lung	Pleomorphic carcinoma	Right bronchial artery embolization, right upper lobectomy, adjuvant ChT	Partial gastrectomy, immunotherapy	Abdominal pain
Maeda et al. ^85^	1	60	F	Lung	SCLC	ChT	N/A	Nausea, vomiting
Struyf et al. ^86^	1	68	M	Lung	Aca	ChT	ChT	Severe epigastric pain
Altintas et al. ^87^	1	55	M	Lung	Aca	ChT	ChT	Epigastric pain, hematemesis, melena
Casella et al. ^88^	1	63	M	Lung	SCLC	Supportive care	Supportive care	Fever, weight loss, epigastric pain, constipation
Ohashi et al. ^89^	1	62	M	Lung	Large cell carcinoma	Right upper lobectomy	ChT	Abdominal pain
Aokage et al. ^90^	2	69	M	Lung - right upper lobe	Pleomorphic carcinoma	Right upper lobectomy, parietal pleura resection	Partial gastrectomy, splenectomy	Fatigue, anemia
		62	M	Lung - left upper lobe	Pleomorphic carcinoma	Left upper lobectomy	Distal gastrectomy, splenectomy	N/A
Katsenos and Archondakis ^91^	1	61	M	Lung - left upper lobe	Aca	ChT	ChT	Upper GIS bleeding
Diem et al. ^92^	1	62	F	Lung - right upper lobe	Aca	N/A	ChT	Epigastric pain
Hu et al. ^93^	1	54	M	Lung	SCC	RT, right middle lobectomy	None (patient refused)	Dysphagia
Koh et al. ^94^	1	46	M	Lung	Pleomorphic carcinoma	Antibiotics	N/A	Abdominal pain, tenderness
Hung et al. ^95^	1	47	M	Lung	SCC	RT, ChT	ChT	Weight loss, dysphagia
Taira et al. ^96^	1	64	M	Lung	Pleomorphic carcinoma, Aca	Left upper lobectomy	ChT	Anemia
Gao et al. ^97^	1	66	M	Lung	SCLC	ChT	ChT, supportive care	Epigastric pain
Kim et al. ^98^	1	68	M	Lung	Poorly differentiated Aca	Left lower lobectomy, posterior segmentectomy right upper lobe (2004), left upper lobe wedge resection (2007), palliative chemotherapy	Palliative ChT	Epigastric pain, dyspepsia
Chen et al. ^99^	1	59	F	Lung	Sarcomatoid carcinoma	Supportive treatment	Supportive treatment	Abdominal pain, anorexia, weight loss
Dong et al. ^100^	1	60	F	Lung	Glomus tumor	N/A	N/A	Hemoptysis, melena, abdominal distension
Kim et al. ^101^	2	66	M	Lung	SCLC	N/A	N/A	Epigastric pain, epigastric tenderness, fatigue
		68	M	Lung	SCLC	N/A	N/A	Hemoptysis, weight loss
Del Rosario et al. ^102^	1	77	F	Lung	Aca	ChT	Palliative care	N/A
Kanthan et al. ^71^	1	75	M	Lung	Aca	N/A	N/A	Epigastric pain, RUQ pain

Aca: adenocarcinoma; SCC: squamous cell carcinoma; RT: radiotherapy; GIS: gastrointestinal; EGD: esophagogastroduodenoscopy; EUS: endoscopic ultrasound; GDS: gastroduodenoscopy; NSCLC: non-small-cell lung cancer; SCLC: small-cell lung cancer; ChT: chemotherapy; RUQ: right upper quadrant; ChT: chemotherapy; USG: ultrasonography; Syn: synchronous; Met: metachronous.

### Malign melanoma

The median age of the 19 patients (seven female, 12 male) was 67, ranging from 28 years to 89 years. In 16 patients, other organ metastases were also discovered in addition to malign melanoma. Overall survival was not mentioned in 10 cases. Two of these cases deceased two and four days after hospital admission respectively, and one patient died after a year. Moreover, one of these patients was alive at five years, and another was alive at six months. Overall survival of three cases is three, 27, and four months, respectively. One of these patients refused treatment, and one of them did not receive treatment. However, immunotherapy was applied to six patients, surgery to five patients, radiotherapy to two patients, and only supportive treatment to three patients. In addition, the treatment of GM was not mentioned in three cases. Table 4 summarizes the findings of included studies regarding malign melanoma. ^103^^–^^121^

**Table 4.** Illustrating data regarding metastasis from malignant melanoma.

First author	No of cases	Age	Sex	Site of primary tumor	Histology type of primary	Treatment of primary	Treatment of metastasis	Clinical presentation of GM
Zhu et al. ^103^	1	36	M	Right plantar	Nodular	Mohs microsurgery	N/A	Anorexia, nausea, vomiting
Yoshimoto et al. ^104^	1	82	F	Fourth left toe	Acral lentiginous	Surgery	Palliative RT	Melena
Okamoto et al. ^105^	1	79	M	Esophagus	Pigmented submucosal tumor-like growth in the esophagus	Nivolumab	Nivolumab	Gross hematuria, weight loss, cough, exertional dyspnea
Cortellini et al. ^106^	1	81	M	N/A	N/A	N/A	N/A	Weakness, hyporexia, anemia
Groudan et al. ^107^	1	66	F	Vulva	N/A	N/A	Palliative RT, immunotherapy	Fatigue, exertional dyspnea, hematemesis, weight loss, nausea
Syed et al. ^108^	1	49	F	Back	N/A	Surgery	Immunotherapy, supportive care, SRS	Anorexia, abdominal pain, fatigue, weight loss, nausea, vomiting
Genova et al. ^109^	1	80	M	Scalp	Lentigo	RT	Immunotherapy	Hypochromic anemia
Wong et al. ^110^	1	81	F	Foot	Acral lentiginous	Amputation, CT	Denied the treatment	Dyspnea, fatigue, anemia
Grander et al. ^111^	1	67	M	Right hypochondrium, back, scalp	Superficial spreading	Surgery	Total gastrectomy, radiosurgery	Melena
Carcelain et al. ^112^	1	65	F	N/A	N/A	Surgery	Surgery	N/S
Lestre et al. ^113^	1	67	M	Lower back	Superficial spreading	Excision, adjuvant immunotherapy	No	N/S
Rana et al. ^114^	1	72	M	N/A	N/A	N/A	N/A	Weight loss, anorexia
Rovere et al. ^115^	1	68	M	N/A	N/A	N/A	Supportive care	N/S
Eivazi-Ziaei et al. ^116^	1	56	M	Right heel-ALM	N/A	Surgery	Supportive care	Epigastric pain
El-Sourani et al. ^117^	1	43	F	Right breast	N/A	Surgery	Sleeve gastrectomy after atypical resection, complete locoregional lymphadenectomy	Melena, anemia
Buissin et al. ^118^	1	63	M	Anorectal	Hyperplastic polyp	Abdominoperineal resection	Supportive care	Tenderness in the RUQ
Bankar et al. ^119^	1	41	F	N/A	N/A	N/A	Surgery	N/S
Mohan et al. ^120^	1	28	M	N/A	N/A	N/A	Temozolomide	Abdominal pain, anorexia, weight loss
Farshad et al. ^121^	1	89	M	Chest wall	N/A	Local excision	Nivolumab	Fatigue, rigors, fever

ALM: acral lentiginous melanoma; ChT: chemotherapy; RUQ: right upper quadrant; EGD: esophagogastroduodenoscopy; NBI: narrow band imaging; RT: radiotherapy; SRS: stereotactic radiosurgery; IHC: immunohistochemistry; CT: computed tomography; EMG: electromyography; Syn: synchronous; Met: metachronous; FDG: fluorodeoxyglucose; PET: positron emission tomography.

### Urogenital cancers

The median age of 20 patients (11 male and nine female) with kidney cancer was 68.5 years old. A total of 11 patients had metastases other than GM. Overall survival was mentioned only in four cases and ranged from two months to one year. One of the 20 patients did not receive any therapy for GM, whereas 13 patients underwent surgical treatment (four endoscopic mucosal resections, nine gastrectomies), four patients had chemotherapy and one patient was treated with radiotherapy. Regarding prostate cancer, the median age of the affected individuals was 67 years old. Concerning the GM treatment four patients received chemotherapy, one patient underwent mucosal resection, and one patient refused treatment. Overall survival was mentioned for three patients ranging from four months to 19 months. All four patients with testis cancer had other metastases and two of them received chemotherapy. One study included bladder cancer without other metastases and the patient was referred to palliative care. Data pertaining to gastric metastases originating from primary urogenital cancers are summarized in Table 5. ^71^^,^^122^^–^^151^

**Table 5.** Illustrating data regarding metastasis from urogenital cancers.

First author	No of cases	Age	Sex	Site of primary tumor	Histology type of primary	Treatment of primary	Treatment of metastasis	Clinical presentation of GM
Tapasak and Mcguirt ^122^	1	77	M	Kidney	RCC	Nephrectomy, ChT	Roux-en-Y gastric bypass	Gastrointestinal bleeding, anemia
Podzolkov et al. ^123^	1	30	M	Testis	Choriocarcinoma	ChT	N/A	Epigastric pain, dyspnea
Koterazawa et al. ^124^	1	70	F	Kidney	RCC	Nephrectomy	Endoscopic submucosal resection	Weight loss
Hakim et al. ^125^	1	86	F	Kidney	RCC	Nephrectomy, ChT	RT	Gastrointestinal bleeding
Yoshida et al. ^126^	1	85	F	Kidney	RCC	Nephrectomy	Endoscopic resection	Anemia, melena
Bernshteyn et al. ^127^	1	68	M	Kidney	RCC	Nephrectomy	N/A	Dyspnea, melena
Weissman et al. ^128^	2	70	M	Kidney	RCC	Nephrectomy	ChT	Dyspepsia, malaise, weight loss
		85	M	Kidney	RCC	Nephrectomy	ChT	Dyspepsia, malaise, weight loss
Chaar et al. ^129^	1	30	M	Testis	Choriocarcinoma	Orchiectomy	ChT (patient refused)	Melena, anemia
Arakawa et al. ^130^	1	80	F	Kidney	RCC	Cht	ChT	Anorexia, pyrexia, malaise
Uehara et al. ^131^	1	73	M	Kidney	RCC	Nephrectomy, ChT	Endoscopic mucosal resection, immunotherapy	Gastric mass
O'Reilly et al. ^132^	1	59	F	Kidney	Clear cell RCC	Nephrectomy	Laparoscopic sleeve gastrectomy	Asymptomatic
Abu Ghanimeh et al. ^133^	1	67	M	Kidney	Clear cell RCC	Nephrectomy	No treatment initiated	Gastrointestinal bleeding
Mazumdar et al. ^134^	1	49	M	Testis	Seminoma	N/A	N/A	Abdominal pain
Barras et al. ^135^	1	53	M	Kidney	RCC	Nephrectomy	Partial gastrectomy	Hematochezia
Riviello et al. ^136^	1	68	M	Kidney	RCC	Nephrectomy	Gastrectomy, ChT	Melena, postural dizziness, weakness
Hong et al. ^137^	1	60	M	Bladder	Clear cell urothelial Ca	ChT, RT	Palliative care	Projectile vomiting
Onitilo et al. ^138^	2	57	M	Prostate	Aca	LHRH agonist	ChT	Weakness, nausea, vomiting, hematemesis
		89	M	Prostate	Aca	LHRH agonist	ChT	Weakness, nausea, vomiting, hematemesis
Tiwari et al. ^139^	1	58	F	Kidney	Clear cell RCC	N/A	Roux-en-Y subtotal gastrectomy	Melena, hematemesis, fatigue
Yodonawa et al. ^140^	1	73	M	Kidney	Leiomyosarcoma	Nephrectomy	Distal gastrectomy	Melena, weakness
Chibbar et al. ^141^	1	69	F	Kidney	Clear cell RCC	Nephrectomy	Endoscopic mucosal resection	Fatigue, lightheadedness, anemia
Sakurai et al. ^142^	1	61	M	Kidney	RCC	Nephrectomy	Partial gastrectomy, ChT	Melena, anemia
Patel et al. ^143^	1	71	M	Prostate	Aca	Surgery, RT	N/A	Weakness, dizziness, anemia
Sharifi et al. ^144^	1	17	F	Kidney	Primitive neuroectodermal	ChT	ChT	Abdominal pain, distention
Greenwald et al. ^145^	1	62	M	Kidney	Clear cell RCC	Nephrectomy	Partial gastrectomy	Testicular pain
Costa et al. ^146^	1	66	F	Kidney	RCC	Nephrectomy	Palliative laparoscopic wedge resection	Anemia
Soe et al. ^147^	1	64	M	Prostate	N/A	LHRH agonist	Palliative care (patient refused chemotherapy)	Anemia, melena
Bhandari and Pant ^148^	1	58	M	Prostate	Aca	LHRH agonist	ChT	Abdominal pain
Lowe et al. ^149^	1	18	M	Testis	Choriocarcinoma	ChT, orchidectomy	ChT	Melena, lethargy, dizziness
Inagaki et al. ^150^	1	75	M	Prostate	Aca	LHRH agonist	Endoscopic mucosal resection, hormone therapy	Epigastric pain
Tavukcu et al. ^151^	1	67	M	Prostate	Mixed 55% ductal 45% acinar	Prostatectomy, RT	Androgen deprivation therapy, ChT	Ascites, vomit
Kanthan et al. ^71^	1	19	M	Testis	Predominantly choriocarcinoma, embryonal Ca	Orchiectomy	Partial gastrectomy聽	Melena, anemia

Ca: carcinoma; ChT: chemotherapy; EUS: endoscopic ultrasound; Aca: adenocarcinoma; RT: radiotherapy; EGD: esophagogastroduodenoscopy; RCC: renal cell carcinoma; LHRH: luteinizing hormone releasing hormone; Syn: synchronous; Met: metachronous.

### Others

The median age of the four patients with Merkel cell carcinoma was 73 years old. Two patients had other metastases in addition to GM. Three patients underwent surgery, chemotherapy, and radiotherapy, whereas one patient was treated with chemotherapy and radiotherapy. One patient with squamous cell carcinoma had other metastases in addition to GM and received chemotherapy and radiotherapy for the primary tumor.

Regarding bone cancers (n=3) one of the patients was 14 years old and stood out as the youngest patient in this group. Concerning the GM therapy, one of the patients with a known treatment underwent surgery and chemotherapy the other received only surgery. In all patients, GM was discovered metachronous. Three studies were included for soft tissue cancer. All three patients had metastases in addition to GM and underwent different types of GM treatment (including radiotherapy, chemotherapy, excision with snare, and cautery). For the thyroid cancer group, the median age was 71 years old. Overall survival (OS) was only mentioned for one patient (2.5 months). Regarding diffuse large B-cell lymphoma (DLBCL) (n=2), patients received chemotherapy for primary cancer and for GM. GM was discovered synchronously. Kovecsi *et al.*, described the only case of GM from adrenocortical carcinoma of the adrenal gland. ^152^ The patient underwent adrenalectomy for primary and total gastrectomy with splenectomy and end-to-side Roux-en-Y esophagojejunal anastomosis for GM. One patient with choriocarcinoma from retroperitoneum underwent chemotherapy for primary cancer and GM. Table 6 summarizes the findings of included studies regarding gastrointestinal cancers. ^151^^–^^171^

**Table 6.** Illustrating data regarding metastasis from other cancers.

First author	No of cases	Age	Sex	Site of primary tumor	Histology type of primary	Treatment of primary	Treatment of Metastasis	Clinical presentation of GM
Kovecsi et al. ^152^	1	71	M	Adrenal gland	Adrenocortical carcinoma	Right adrenalectomy	Total gastrectomy, splenectomy, with end-to-side Roux-en-Y eso-jejunal anastomosis	Weight loss, epigastric pain, vomiting, fatigue
Koti et al. ^153^	1	14	F	Bone	Ewing sarcoma	ChT, local excision	ChT, total gastrectomy, RT	Abdominal mass, low-grade fever, weight loss
Dodis et al. ^154^	1	72	F	Bone	Ewing sarcoma	Total knee replacements, RT, ChT	N/A	Anemia
Urakawa et al. ^155^	1	73	M	Bone	Osteosarcoma	ChT, surgery	Partial gastrectomy	Anemia, hematemesis
Shibuya et al. ^156^	1	27	M	Extragonadal retroperitoneal	Choriocarcinoma	Cht	ChT	Abdominal pain, melena, vomiting
Tarangelo et al. ^157^	1	65	M	Head, neck	SCC	Cht, RT, robotic excision	N/A	Melenic bowel movements
Kamihara et al. ^158^	1	70	M	Lymph nodes	DLBC	R-CHOP ChT	R-CHOP ChT	N/A
Zepeda-Gomez et al. ^159^	1	39	F	Lymph nodes	DLBC	ChT, omeprazole	ChT	Melena, weight loss, retroperitoneal mass
Teh et al. ^160^	1	37	F	Oropharynx	SCC	Surgery, adjuvant RT	Palliative RT	Weight loss, LUQ pain, melena
Elkafrawy et al. ^161^	1	67	M	Skin	MCC	Surgery, consolidative	Atezolizumab, RT	Melena
Ha et al. ^162^	1	82	M	Skin	MCC	Surgery, RT	No	Anorexia, weight loss
Idowu et al. ^163^	1	79	F	Skin	MCC	Surgery, ChT, RT	N/A	Anemia
Parikh et al. ^164^	1	60	M	Skin	MCC	ChT, RT	ChT	Maroon colored stools
Subramanian et al. ^165^	1	62	M	Soft tissue	Leiomyosarcoma	Surgery, RT	RT, ChT	Melena, abdominal pain, nausea, vomiting
Dent et al. ^166^	1	60	M	Soft tissue	Sarcoma	Surgery	Remove with snare and cautery	Upper abdominal pain, melena
Samuel et al. ^167^	1	56	M	Soft tissue	Synovial sarcoma	Surgery, RT	Doxorubicin	N/A
Thorburn et al. ^168^	1	56	M	Supraglottic larynx, hypopharynx	Advanced SCC	Surgery, tracheostomy, radical RT	N/A	Anemia, hematemesis
Fuladi et al. ^169^	1	71	F	Thyroid	Anaplastic carcinoma	Total thyroidectomy, left modified radical neck dissection, RT	N/A	Nausea, vomiting
Ayaz et al. ^170^	1	72	M	Thyroid	Anaplastic carcinoma	N/A	N/A	Melena
Karrasch et al. ^171^	1	53	F	Thyroid	Medullary thyroid cancer	Complete thyroidectomy ChT	N/A	Fatigue, anorexia, epigastric pain radiating to the back

SCC: squamous cell carcinoma; MCC: merkel cell carcinoma; RT: radiotherapy; EGD: esophagogastroduodenoscopy; DLBC: diffuse large B cell; ChT: chemotherapy; LUQ: left upper quadrant; Met: metachronous; EGD: esophagogastroduodenoscopy; FDG: fluorodeoxyglucose; PET: positron emission tomography; Syn: synchronous.

## Discussion

Gastric metastases are uncommon and give information about the progressed stage of malignant disease, with a reported incidence of 0.2-0.7% based on clinical and autopsy findings. ^1^ Furthermore, metastasis to the stomach frequently indicates short survival. These metastases are observed rarely due to clinical problems regarding their diagnosis and treatment. ^2^ Progressively, with improvements in prognosis for cancer patients, metastatic tumors in the stomach are being detected more frequently. ^1^ There are several symptoms of gastric metastases, such as abdominal pain, diarrhea, nausea, vomiting, weight loss, and dyspepsia. The most preferred treatment method for gastric metastasis is surgical resection of the tumor. Also, chemotherapy is the most applied alternative option.

This systematic review has a few potential limitations that need to be mentioned. As 172 case reports were screened in the systematic review from different journals the heterogeneity was inevitable. Some articles missed important information such as complete follow-up or clinical information. Moreover, since all of the included articles were case reports, quality or bias assessment could not be performed.

### Gynecologic cancer

Gastric metastasis mainly occurs due to breast cancer. Both ovarian and uterine metastases are distinctly less frequent. ^38^ Invasive lobular carcinoma is the type with the highest affinity to the digestive system with an incidence of 4.5% compared to 0.2% in ductal carcinoma. ^26^ Breast cancer metastases to the gastrointestinal tract are rare, with a median time interval from the diagnosis of the primary tumor to metastasis up to seven years. ^21^ The longest disease-free interval is 22 years after the initial diagnosis 17 of 24. ^10^ Some metastatic tumors may have a similar presentation as primary gastric cancer. ^38^ The detailed immunohistochemical analysis will allow the most accurate diagnosis to differentiate between primary gastric cancer and gastric metastasis from breast cancer. ^26^ Most gastric metastatic breast cancers are estrogen receptor (ER)-positive, progesterone receptor (PR)-positive/negative, and human epidermal growth factor receptor (HER2)-negative. However, in primary gastric adenocarcinoma, ER and PR can be positively expressed in 20-28% of patients. ^19^ In a few cases, metastatic breast cancer is negative for ER and PR, so a diagnosis cannot be made based on these two investigations alone. ^59^ ER and PR can be used as markers; however, they are not always suitable diagnostic markers to confirm if a tumor has originated. ^11^ Treatment of gastrointestinal metastases from breast cancer is discussed frequently in the literature. Systemic therapy is the first option. ^36^ The effective rate of systemic treatment is about 46%. ^60^ Surgical treatment may have a role in palliative treatment. ^34^ Surgical treatment is considered in cases with obstruction or bleeding. ^36^ Metastasis to the gastrointestinal tract can be the first presentation of breast cancer, therefore it is imperative to consider the possibility of breast cancer metastasis to the gastrointestinal metastasis. ^26^^,^^46^

### Gastrointestinal cancer

Among cancers that metastasize to the stomach, gastrointestinal system cancers are encountered in a minority. Gastric metastases gave unspecific findings, such as anemia, bleeding, and pain. Pancreas, liver, and colon account for the majority of primary cancers. Nine of the cases had other metastases in addition to gastric ones. Since pancreatic cancers are usually caught at an advanced stage, the chances of surgical treatment and their response to treatment are low. We see that three of five patients died within a year. ^2^^,^^6^^,^^8^ Among these cases, the prognosis of pancreatic head cancers was worse than body and tail cancers.

### Lung cancer

In fact, lung cancer is the most mortal type of all cancers. However, the stomach is not a common site for primary lung cancers’ metastases, especially compared with brain, liver, adrenal glands, and bones. ^74^ Yet, the expected lifespan after diagnosis of metastasis is found to be relatively low. The median survival time was four months (average 6.8 months) among 16 cases who died. On the other hand, data showed that endoscopy is the gold standard in diagnosis. In addition, pathology and immunohistochemistry are considered important factors to differentiate gastric metastases from primary cancers. ^81^ Regarding the treatment of gastric metastases of the pulmonary origin, although non-invasive chemotherapy treatments were the most common choice, patients who received surgery, particularly partial gastrectomy, but also esophagogastrostomy and laparotomy, tended to have relatively much longer survival time. ^77^^,^^90^^,^^95^ However, this conclusion is not definitive, since in some cases surgeries may be avoided when the patient’s condition is extremely severe and the number of cases with given surgical treatments is scarce. So the potential benefit of surgeries to the expected lifespan of the patients needs further investigation.

### Malign melanoma

Although melanoma accounts for only 5% of cutaneous malignancies, it makes up nearly 75% of skin cancer- related deaths. ^103^^,^^107^ Malignant melanoma ranks as the most common metastatic tumor of the gastrointestinal (GI) tract. ^103^^,^^110^^,^^120^ It takes an average of 52 months for a primary cutaneous melanoma to spread to the gastrointestinal tract. ^107^^,^^110^ Only 1-4% of patients with malignant melanoma deceased before gastrointestinal metastases are diagnosed. On the other hand, GI tract metastasis was observed in more than 60% of melanoma patients by autopsy. ^103^^,^^110^^,^^111^^,^^114^^,^^121^ The most commonly involved sites include the small and large bowels and rectum; however, gastric metastasis is a rare case ^110^^,^^111^^,^^117^^,^^119^^,^^121^ due to the non-specificity of its symptoms, such as epigastric discomfort, nausea, vomiting, weight loss, hematemesis, and melena. ^103^^,^^107^^,^^110^^,^^111^^,^^114^^,^^117^^,^^121^ The average survival is four to six months. ^103^^,^^107^^,^^121^ Endoscopy is an effective method for detecting melanoma metastases due to pigmentation, which can then be confirmed by histology and immunohistochemistry. ^114^^,^^121^ Treatment options include surgical resection, immunotherapy, chemotherapy, and targeted therapy. If a patient is symptomatic, surgical excision can be a palliative technique that can also prolong survival. ^103^^,^^121^

### Urogenital cancer

Regarding urogenital metastases GM is uncommon and the incidence is reported to vary between 0.2% and 0.7%. ^123^ The most common clinical presentations are gastrointestinal bleeding (melena and hematemesis), anemia, and malaise. Whereas two patients had no symptoms associated with the gastrointestinal system. ^131^^,^^140^ Esophagogastroduodenoscopy is often necessary for diagnosis and localized treatment. ^131^ The presence of gastric metastases is considered an important indicator of advanced disease. ^149^ Treatment options varied depending on the stage of the metastasis including endoscopic resection, partial or total gastrectomy, chemotherapy, and palliative care. Even though overall survival seems to be longer in patients who underwent surgery, the main reason for this may be that these patients have early-stage diseases suitable for surgery. Therefore, treatment options should be decided upon the stage of the disease and the general well-being of the patient.

### Others

The most common symptoms, in terms of frequency, are melena, abdominal pain, vomiting, weight loss, anemia, fatigue, and loss of appetite. Gastrointestinal endoscopy plays an important role in the diagnosis of GM if suspected. ^162^ Tumor seeding after endoscopic gastrostomy tube replacement was observed in two cases. ^161^^,^^169^ Even though surgery is the frequent treatment for solid organ cancer metastasis, chemotherapy is the chosen treatment for DLBCL, skin cancer, and sarcoma. Overall survival was only mentioned for four cases; therefore, it is difficult to comment on which treatment method is more beneficial. Metastasis to the stomach is not reported frequently. Thus, determining the prognosis and planning the treatment based on scientific evidence seems to be problematic for clinicians.

In conclusion, among 172 case reports reviewed, resection surgery was performed the most for treatment and was sometimes combined with chemotherapy and immunotherapy. However, the literature regarding the management of patients with secondary gastric cancer is limited. Therefore, further multi-centric research to reach a consensus about what type of treatment has the best outcomes for patients with gastric metastases is needed.

## Data Availability

All data underlying the results are available as part of the article and no additional source data are required. OSF: Tables for ‘Metastasis to the stomach: a systematic review’. https://doi.org/10.17605/OSF.IO/Y4QD5. ^172^ OSF: PRISMA checklist for ‘Metastasis to the stomach: a systematic review’. https://doi.org/10.17605/OSF.IO/Y4QD5. ^172^ Data are available under the terms of the Creative Commons Zero “No rights reserved” data waiver (CC0 1.0 Public domain dedication).

## References

[1] Namikawa T Munekage E Ogawa M : Clinical presentation and treatment of gastric metastasis from other malignancies of solid organs. *Biomed Rep.* 2017;7:159–162. 10.3892/br.2017.943 28804629 PMC5526074

[2] Fousekis FS Tepelenis K Stefanou SK : Gastric metastasis from breast cancer presenting as dysphagia. *J Surg Case Rep.* 2022;2022. 10.1093/jscr/rjac080 35308257 PMC8929746

[3] Watanabe Y Horimoto Y Takahashi Y : A gastric metastatic lesion that resembled early-stage gastric cancer on endoscopy during treatment for recurrent breast cancer: a case report. *Case Rep Oncol.* 2021;14:1719–1724. 10.1159/000520828 35082631 PMC8738911

[4] Husain S Isa M Almarzooq R : A rare metastatic site of invasive lobular breast carcinoma: a case report. *Case Rep Surg.* 2021;2021. 10.1155/2021/9922296 34853710 PMC8629669

[5] Zhang LL Rong XC Yuan L : Breast cancer with an initial gastrointestinal presentation: a case report and literature review. *Am J Transl Res.* 2021;13:13147–13155. 34956535 PMC8661181

[6] Jabi R Karich N Ouryemchi M : Hematemesis: an exceptional method of revealing gastric metastasis from an unknown breast cancer. *Cureus.* 2021;13:e18987. 10.7759/cureus.18987 34820242 PMC8607324

[7] Johnson L Ford R Tofteland N : Gastric metastasis of breast cancer found on routine esophageal variceal screening. *Kans J Med.* 2021;14:162. 10.17161/kjm.vol1415032 34178249 PMC8222103

[8] Okamoto T Suzuki H Fukuda K : Simultaneous gastric cancer and breast cancer metastases to the stomach with lymph node collision tumor: a case report. *BMC Gastroenterol.* 2021;21:240. 10.1186/s12876-021-01823-4 34034653 PMC8146653

[9] Nehmeh WA Derienne J El Khoury L : A 58-year-old woman with acute gastric perforation due to metastatic ductal carcinoma 18 years following bilateral mastectomy for invasive ductal carcinoma of the breast. *Am J Case Rep.* 2021;22:e927094. 10.12659/AJCR.927094 33828068 PMC8042419

[10] Hanafiah M Sidek S Low SF : A case of infiltrating lobular carcinoma of the breast with gastric metastasis 22 years after initial surgery. *Acta Clin Croat.* 2021;60:136–140. 10.20471/acc.2021.60.01.20 34588734 PMC8305346

[11] Teixeira S Sousa C Castro M : Gastric metastases from invasive lobular carcinoma of the breast: case report. *Radiol Case Rep.* 2021;16:372–376. 10.1016/j.radcr.2020.11.051 33318776 PMC7726484

[12] Kutasovic JR McCart Reed AE Sokolova A : Phenotypic drift in metastatic progression of breast cancer: a case report with histologically heterogeneous lesions that are clonally related. *Clin Case Rep.* 2020;8:2725–2731. 10.1002/ccr3.3257 33363813 PMC7752647

[13] Abdallah H Elwy A Alsayed A : Metastatic breast lobular carcinoma to unusual sites: a report of three cases and review of literature. *J Med Cases.* 2020;11:292–295. 10.14740/jmc3538 34434416 PMC8383673

[14] Liu HP Chang WY Hsu CW : A giant malignant phyllodes tumor of 19 of 24breast post mastectomy with metastasis to stomach manifesting as anemia: a case report and review of literature. *BMC Surg.* 2020;20:187. 10.1186/s12893-020-00846-0 32799838 PMC7430829

[15] Tang T Zhang L Li C : Gastric and adrenal metastasis from breast cancer: case report and review of literature. *Medicine (Baltimore).* 2020;99:e18812. 10.1097/MD.0000000000018812 32011488 PMC7220226

[16] De Gruttola I Adil MT D’Souza L : Perforated gastric carcinomatosis following invasive lobular cancer of the breast. *Clin Case Rep.* 2019;7:999–1002. 10.1002/ccr3.2116 31110734 PMC6509928

[17] Mohy-Ud-Din N Patek B Dhawan M : Unusual presentation of gastric outlet obstruction due to breast cancer metastasis: a case report. *Cureus.* 2019;11:e4533. 10.7759/cureus.4533 31263641 PMC6592458

[18] Güler SA Şimşek T Pösteki G : A very rare reason for gastric perforation, caused by gastric metastasis of breast cancer: case presentation. *Eur J Breast Health.* 2019;15:59–62. 10.5152/ejbh.2018.4285 30816356 PMC6385715

[19] Cui M Zhang X Harpaz N : Isolated gastric metastasis of endometrial adenocarcinoma: first case report and review of pertinent literature. *Gastroenterology Res.* 2018;11:422–425. 10.14740/gr1071w 30627266 PMC6306114

[20] Asmar N Rey JF Sattonnet C : Gastric metastasis mimicking linitis plastica 20 years after primary breast cancer. A case report. *J Gastrointestin Liver Dis.* 2018;27:469–471. 10.15403/jgld.2014.1121.274.gas 30574631

[21] Klair JS Soota K Vidholia A : Gastric metastasis of an ovarian granulosa cell tumor diagnosed in a patient with worsening reflux. *ACG Case Rep J.* 2018;5:e791–e792. 10.14309/crj.2018.79 30465009 PMC6224866

[22] Yang S : Gastric metastasis of ovarian serous cystadenocarcinoma. *Int Med Case Rep J.* 2018;11:201–204. 10.2147/IMCRJ.S171985 30233254 PMC6130277

[23] Bushan K Kammar P Singh C : Infiltrating lobular breast cancer presenting as isolated gastric metastasis: a case report. *Indian J Surg Oncol.* 2018;9:318–322. 10.1007/s13193-017-0705-7 30287990 PMC6154363

[24] Zhang B Copur-Dahi N Kalmaz D : Gastrointestinal manifestations of breast cancer metastasis. *Dig Dis Sci.* 2014;59:2344–2346. 10.1007/s10620-014-3155-x 24748230 PMC6658174

[25] Jin X Tang H Chen H : Case report: metastatic signet-ring-cell carcinoma of the bladder from breast invasive lobular carcinoma detected by computed tomography. *Front Oncol.* 2022;12. 10.3389/fonc.2022.835487 35252006 PMC8888881

[26] Buka D Dvořák J Richter I : Gastric and colorectal metastases of lobular breast carcinoma: a case report. *Acta Med (Hradec Kralove).* 2016;59:18–21. 10.14712/18059694.2016.50 27131352

[27] Dória MT Maesaka JY Martins SN : Gastric metastasis as the first manifestation of an invasive lobular carcinoma of the breast. *Autops Case Rep.* 2015;5:49–53. 10.4322/acr.2015.018 PMC4636107 26558248

[28] Hwangbo S Kwon OK Chung HY : Improved survival of a patient with gastric and other multiple metastases from ovarian cancer by multimodal treatment: a case report. *J Gastric Cancer.* 2015;15:218–221. 10.5230/jgc.2015.15.3.218 26468421 PMC4604338

[29] Rachan Shetty KS Challa VR Lakshmaiah KC : Gastric metastases from breast cancer: a report of two cases and review of literature. *J Cancer Res Ther.* 2015;11:660. 10.4103/0973-1482.139523 26458672

[30] Geredeli C Dogru O Omeroglu E : Gastric metastasis of triple negative invasive lobular carcinoma. *Rare Tumors.* 2015;7:57–59. 10.4081/rt.2015.5764 26266010 PMC4508641

[31] Kim EY Park CH Jung ES : Gastric metastasis from ovarian cancer presenting as a submucosal tumor: a case report. *J Gastric Cancer.* 2014;14:138–141. 10.5230/jgc.2014.14.2.138 25061543 PMC4105380

[32] Fernandes GS Corrêa TS Carvalho EP : Gastric and endobronchial metastases in a case of lobular breast cancer. *Case Rep Oncol.* 2013;6:555–560. 10.1159/000356564 24348393 PMC3843932

[33] Moldovan B Banu E Pocreaţă D : Gastric metastasis of cervix uteri carcinoma, rare cause of lower gastric stenosis. *Chirurgia (Bucur).* 2012;107:816–820. 23294965

[34] Zhou JJ Miao XY : Gastric metastasis from ovarian carcinoma: a case report and literature review. *World J Gastroenterol.* 2012;18:6341–6344. 10.3748/wjg.v18.i43.6341 23180959 PMC3501787

[35] Critchley AC Harvey J Carr M : Synchronous gastric and colonic metastases of invasive lobular breast carcinoma: case report and review of the literature. *Ann R Coll Surg Engl.* 2011;93:e49–e50. 10.1308/147870811X582800 21943448 PMC5827216

[36] Hara F Kiyoto S Takabatake D : Metastatic breast cancer to the stomach resembling early gastric cancer. *Case Rep Oncol.* 2010;3:142–147. 10.1159/000313923 20740187 PMC2919990

[37] Ciulla A Castronovo G Tomasello G : Gastric metastases originating from occult breast lobular carcinoma: diagnostic and therapeutic problems. *World J Surg Oncol.* 2008;6. 10.1186/1477-7819-6-78 18652707 PMC2525652

[38] Jones GE Strauss DC Forshaw MJ : Breast cancer metastasis to the stomach may mimic primary gastric cancer: report of two cases and review of literature. *World J Surg Oncol.* 2007;5. 10.1186/1477-7819-5-75 PMC1937002 17620117

[39] Yim H Jin YM Shim C : Gastric metastasis of mammary signet ring cell carcinoma - a differential diagnosis with primary gastric signet ring cell carcinoma. *J Korean Med Sci.* 1997;12:256–261. 10.3346/jkms.1997.12.3.256 9250925 PMC3054281

[40] Wong CS Gumber A Kiruparan P : Gastric perforation secondary to metastasis from breast cancer. *BMJ Case Rep.* 2016;2016:bcr2016214865. 10.1136/bcr-2016-214865 27435841 PMC4964250

[41] Ricciuti B Leonardi GC Ravaioli N : Ductal breast carcinoma metastatic to the stomach resembling primary linitis plastica in a male patient. *J Breast Cancer.* 2016;19:324–329. 10.4048/jbc.2016.19.3.324 27721883 PMC5053318

[42] Fernandes G Batista Bugiato Faria LD Assis Pereira I de : Gastric metastasis of breast cancer: a case series. *Rare Tumors.* 2016;8. 10.4081/rt.2016.6305 27746881 PMC5064297

[43] Zullo A Balsamo G Lorenzetti R : Gastric metastases from gynaecologic tumors: case reports and review of the literature. *Ann Transl Med.* 2016;4:483. 10.21037/atm.2016.12.51 28149845 PMC5233532

[44] Guzmán JC Espinosa J Cervera R : Gastric and colon metastasis from breast cancer: case report, review of the literature, and possible underlying mechanisms. *Breast Cancer (Dove Med Press).* 2017;9:1–7. 10.2147/BCTT.S79506 28096693 PMC5207330

[45] Mizuguchi K Minato H Yoshida I : Solitary gastric metastasis from a stage IA serous ovarian carcinoma: a case report with literature review. *Intern Med.* 2017;56:915–919. 10.2169/internalmedicine.56.7784 28420839 PMC5465407

[46] Jmour O Belaïd A Mghirbi F : Gastric metastasis of bilateral breast cancer. *J Gastrointest Oncol.* 2017;8:E16–E20. 10.21037/jgo.2016.10.03 28280631 PMC5334049

[47] Yim K Ro SM Lee J : Breast cancer metastasizing to the stomach mimicking primary gastric cancer: a case report. *World J Gastroenterol.* 2017;23:2251–2257. 10.3748/wjg.v23.i12.2251 28405154 PMC5374138

[48] Choi DI Chi HS Lee SH : A rare case of phyllodes tumor metastasis to the stomach presenting as anemia. *Cancer Res Treat.* 2017;49:846–849. 10.4143/crt.2016.188 27586673 PMC5512377

[49] Khan I Malik R Khan A : Breast cancer metastases to the gastrointestinal tract presenting with anemia and intra-abdominal bleed. *Cureus.* 2017;9. 10.7759/cureus.1429 PMC5587403 28924517

[50] Mullally WJ O’Súilleabháin CB Brady C : Vinorelbine induced perforation of a metastatic gastric lesion. *Ir J Med Sci.* 2017;186:571–575. 10.1007/s11845-016-1536-1 28039597 PMC5550518

[51] Kliiger J Gorbaty M : Metastasis to the pancreas and stomach from a breast cancer primary: a case report. *J Community Hosp Intern Med Perspect.* 2017;7:234–237. 10.1080/20009666.2017.1369379 29046750 PMC5637649

[52] Antonini F Laterza L Fuccio L : Gastric metastasis from ovarian adenocarcinoma presenting as a subepithelial tumor and diagnosed by endoscopic ultrasound-guided tissue acquisition. *World J Gastrointest Oncol.* 2017;9:452–456. 10.4251/wjgo.v9.i11.452 29204254 PMC5700387

[53] Kono M Nagami Y Ominami M : A metastatic gastric tumor from ovarian cancer. *Intern Med.* 2018;57:345–349. 10.2169/internalmedicine.9147-17 29093397 PMC5827314

[54] Kim DH Son SM Choi YJ : Gastric metastasis from invasive lobular breast cancer, mimicking primary gastric cancer: a case report. *Medicine (Baltimore).* 2018;97:e0258. 10.1097/MD.0000000000010258 29595684 PMC5895432

[55] Woo J Lee JH Lee KE : Gastric metastasis as the first presentation one year before diagnosis of primary breast cancer. *Am J Case Rep.* 2018;19:354–359. 10.12659/ajcr.908039 29576606 PMC5884313

[56] Ulmer LL Cormier I Jha LK : Use of endoscopic ultrasound in a diagnostic dilemma: metastatic breast cancer to the stomach. *Case Rep Gastrointest Med.* 2018;2018:1–3. 10.1155/2018/2820352 29850292 PMC5925204

[57] Iwai N Okuda T Harada T : Gastric metastasis from colorectal cancer mimicking a submucosal tumor. *Case Rep Gastroenterol.* 2020;14:338–345. 10.1159/000508414 32884508 PMC7443688

[58] Yang J Yuan Y Zhang S : Gastric metastasis from pancreatic cancer characterized by mucosal erosion: a case report and literature review. *J Int Med Res.* 2021;49:030006052110037. 10.1177/03000605211003759 33840245 PMC8044569

[59] Lee WY Lee HK : A sequentially metastatic gastric and jejunal cancer originating from colon cancer: a case report. *Int J Surg Case Rep.* 2020;71:172–175. 10.1016/j.ijscr.2020.05.009 32470913 PMC7260398

[60] Rothermel LD Strosberg C Centeno BA : Case report of isolated gastric metastasis of pancreatic cancer from a diagnostic biopsy: management of a rare oncologic entity. *Cancer Control.* 2020;27:107327482090404. 10.1177/1073274820904042 32107943 PMC7053786

[61] Terashima S Watanabe S Kogure M : Long-term survival after resection of a gastric metastasis from transverse colon cancer: a case report. *Fukushima J Med Sci.* 2019;65:37–42. 10.5387/fms.2018-24 31167978 PMC6760918

[62] Sasajima J Okamoto K Taniguchi M : Hematogenous gastric metastasis of pancreatic cancer. *Case Rep Gastroenterol.* 2016;10:75–80. 10.1159/000444249 27403106 PMC4929381

[63] Tomonari A Katanuma A Matsumori T : Resected tumor seeding in stomach wall due to endoscopic ultrasonography-guided fine needle aspiration of pancreatic adenocarcinoma. *World J Gastroenterol.* 2015;21:8458–8461. 10.3748/wjg.v21.i27.8458 26217099 PMC4507117

[64] Adachi K : Primary squamous cell carcinoma of the pancreas: a case report. *JOP.* 2011;12:181–184. 21386649

[65] Nakazawa N Fukuchi M Sakurai S : Mucosal esophageal squamous cell carcinoma with intramural gastric metastasis invading liver and pancreas: a case report. *Int Surg* 2014;99:458–462. 10.9738/INTSURG-D-13-00069.1 25058784 PMC4114380

[66] Abouzied MM Fathala A AlMuhaideb A : Gastric wall metastases from hepatocellular carcinoma: case report and review of the literature. *Radiol Case Rep.* 2021;16:550–554. 10.1016/j.radcr.2020.12.047 33384755 PMC7770481

[67] Ito S Takahashi Y Yamada T : Intrahepatic cholangiocarcinoma with gastric infiltration misdiagnosed as gastric submucosal tumor. *J Surg Case Rep.* 2020;2020. 10.1093/jscr/rjaa359 33214863 PMC7655014

[68] Imai M Ishikawa T Okoshi M : Hemorrhagic gastric metastasis from hepatocellular carcinoma successfully treated using coil embolization of the left gastric artery. *Intern Med.* 2019;58:2179–2183. 10.2169/internalmedicine.2172-18 30996163 PMC6709331

[69] Kim R Song J Kim SB : Concurrent hepatocellular carcinoma metastasis to stomach, colon, and brain: A case report. *World J Clin Cases.* 2020;8:3534–3541. 10.12998/wjcc.v8.i16.3534 32913860 PMC7457111

[70] Peng L Yu K Li Y : Gastric metastasis of recurrent hepatocellular carcinoma: a case report and literature review. *J Cancer Res Ther.* 2018;14:S1230–S1232. 10.4103/0973-1482.199379 30539878

[71] Kanthan R Sharanowski K Senger JL : Uncommon mucosal metastases to the stomach. *World J Surg Oncol.* 2009;7. 10.1186/1477-7819-7-62 19650900 PMC2734526

[72] Wang JK Su F Ma WJ : Primary malignant melanoma of the gallbladder with multiple metastases: a case report. *Medicine (Baltimore).* 2017;96:e8793. 10.1097/MD.0000000000008793 29145341 PMC5704886

[73] Catalano M Marini A Ferrari K : Gastric and colonic metastasis from NSCLC: a very unusual case report. *Medicine (Baltimore).* 2022;101:e28249. 10.1097/MD.0000000000028249 35029172 PMC8758018

[74] Shih-Chun C Shih-Chiang H Chun-Yi T : Non-small cell lung cancer with gastric metastasis and repeated gastrointestinal bleeding: a rare case report and literature review. *Thorac Cancer.* 2021;12:560–563. 10.1111/1759-7714.13815 33403816 PMC7882379

[75] Das Majumdar SK Mahapatra BR Muraleedharan A : Response to immunotherapy in adenocarcinoma lung with gastric metastasis: a rare case report and review of literature. *Cureus.* 2021;13:e19790. 10.7759/cureus.19790 34956782 PMC8693547

[76] Liu J Xia L Peng Y : Gastric metastasis and transformation of primary lung adenocarcinoma to small cell cancer after acquired resistance to epidermal growth factor receptor tyrosine kinase inhibitors: a case report. *Medicine (Baltimore).* 2021;100:e27289. 10.1097/MD.0000000000027289 34596125 PMC8483845

[77] Nemoto M Prasoon P Ichikawa H : Primary lung squamous cell carcinoma and its association with gastric metastasis: a case report and literature review. *Thorac Cancer.* 2020;11:1708–1711. 10.1111/1759-7714.13410 32212371 PMC7262906

[78] He Y Cui Y Duan X : Primary lung squamous cell carcinoma with gastric metastasis: a case report. *Thorac Cancer.* 2019;10:373–377. 10.1111/1759-7714.12940 30561123 PMC6360227

[79] Yang X Chen R Wu C : Mutational analysis on gastric, duodenal, bone, and mediastinal lymph node metastases and blood from a case of primary lung adenocarcinoma. *Onco Targets Ther.* 2018;11:4029–4034. 10.2147/OTT.S167602 30034242 PMC6049053

[80] Li X Li S Ma Z : Multiple gastrointestinal metastases of squamous-cell lung cancer: a case report. *Medicine (Baltimore).* 2018;97:e11027. 10.1097/MD.0000000000011027 29901596 PMC6023823

[81] Bhardwaj R Bhardwaj G Gautam A : Upper gastrointestinal bleed as a manifestation of poorly differentiated metastatic squamous cell carcinoma of the lung. *J Clin Diagn Res.* 2017;11:OD13–OD14. 10.7860/JCDR/2017/27040.10090 28764229 PMC5535421

[82] Badipatla KR Yadavalli N Vakde T : Lung cancer metastasis to the gastrointestinal system: an enigmatic occurrence. *World J Gastrointest Oncol.* 2017;9:129–134. 10.4251/wjgo.v9.i3.129 28344748 PMC5348628

[83] Qasrawi A Abu Ghanimeh M Albadarin S : Gastric metastases from lung adenocarcinoma causing gastrointestinal bleeding. *ACG Case Rep J.* 2017;4:e25. 10.14309/crj.2017.25 28286791 PMC5340659

[84] Kim J Thomashow B Saqi A : Pulmonary cavitary lesion and haemoptysis: rare aetiology on biopsy. *BMJ Case Rep.* 2016;2016:bcr2016216683. 10.1136/bcr-2016-216683 27507695 PMC4986068

[85] Maeda J Miyake M Tokita K : Small cell lung cancer with extensive cutaneous and gastric metastases. *Intern Med.* 1992;31:1325–1328. 10.2169/internalmedicine.31.1325 1338292

[86] Struyf N Lacor P Van den Weyngaert D : Gastric metastases from lung carcinoma. *Ann Oncol.* 1991;2:694–695. 10.1016/S0923-7534(20)30677-3 1660300

[87] Altintas E Sezgin O Uyar B : Acute upper gastrointestinal bleeding due to metastatic lung cancer: an unusual case. *Yonsei Med J.* 2006;47:276–277. 10.3349/ymj.2006.47.2.276 16642561 PMC2687641

[88] Casella G Di Bella C Cambareri AR : Gastric metastasis by lung small cell carcinoma. *World J Gastroenterol.* 2006;12:4096–4097. 10.3748/wjg.v12.i25.4096 16810769 PMC4087731

[89] Ohashi K Kiura K Takigawa N : Successful treatment of a patient with gastric and duodenal metastases from large cell carcinoma of the lung with carboplatin and gemcitabine. *Anticancer Res.* 2006;26:4695–4696. 17214328

[90] Aokage K Yoshida J Ishii G : Long-term survival in two cases of resected gastric metastasis of pulmonary pleomorphic carcinoma. *J Thorac Oncol.* 2008;3:796–799. 10.1097/JTO.0b013e31817c925c 18594328

[91] Katsenos S Archondakis S : Solitary gastric metastasis from primary lung adenocarcinoma: a rare site of extra-thoracic metastatic disease. *J Gastrointest Oncol.* 2013;4:E11–E15. 10.3978/j.issn.2078-6891.2012.057 23730522 PMC3635193

[92] Diem S Früh M Rodriguez R : EML4-ALK-positive pulmonary adenocarcinoma with an unusual metastatic pattern: a case report. *Case Rep Oncol.* 2013;6:316–319. 10.1159/000352086 23898275 PMC3725025

[93] Hu JB Zhu YH Jin M : Gastric and duodenal squamous cell carcinoma: metastatic or primary? *World J Surg Oncol.* 2013;11. 10.1186/1477-7819-11-204 23957943 PMC3751751

[94] Koh H Chiyotani A Tokuda T : Pleomorphic carcinoma showing rapid growth, multiple metastases, and intestinal perforation. *Ann Thorac Cardiovasc Surg.* 2014;20:669–673. 10.5761/atcs.cr.13-00167 24492166

[95] Hung TI Chu KE Chou YH : Gastric metastasis of lung cancer mimicking an adrenal tumor. *Case Rep Gastroenterol.* 2014;8:77–81. 10.1159/000360845 24748862 PMC3985806

[96] Taira N Kawabata T Ichi T : A case of synchronous double primary lung cancer presenting with pleomorphic carcinoma and adenocarcinoma. *Am J Case Rep.* 2014;15:576–579. 10.12659/AJCR.892339 25553415 PMC4281025

[97] Gao S Hu XD Wang SZ : Gastric metastasis from small cell lung cancer: a case report. *World J Gastroenterol.* 2015;21:1684–1688. 10.3748/wjg.v21.i5.1684 25663792 PMC4316115

[98] Kim MJ Hong JH Park ES : Gastric metastasis from primary lung adenocarcinoma mimicking primary gastric cancer. *World J Gastrointest Oncol.* 2015;7:12–16. 10.4251/wjgo.v7.i3.12 25780510 PMC4357873

[99] Chen CH Chen WM Tung SY : Gastrointestinal metastasis from primary sarcomatoid carcinoma of the lung: a case report and review of the literature. *World J Surg Oncol.* 2015;13:174. 10.1186/s12957-015-0599-1 25947890 PMC4440284

[100] Dong LL Chen EG Sheikh IS : Malignant glomus tumor of the lung with multiorgan metastases: case report and literature review. *Onco Targets Ther.* 2015;8:1909–1914. 10.2147/OTT.S89396 26251614 PMC4524584

[101] Kim HS Jang WI Hong HS : Metastatic involvement of the stomach secondary to lung carcinoma. *J Korean Med Sci.* 1993;8:24–29. 10.3346/jkms.1993.8.1.24 8393680 PMC3053845

[102] Del Rosario M Tsai H : Not all gastric masses are gastric cancer. *BMJ Case Rep.* 2016;2016:bcr2015213535. 10.1136/bcr-2015-213535 26976833 PMC4800203

[103] Zhu M Zhang DY Zhang GJ : Amelanotic metastatic gastric malignant melanoma: a case report. *Anti-Cancer Drugs.* 2022;33:e808–e812. 10.1097/CAD.0000000000001227 34459456 PMC8670341

[104] Yoshimoto T Okamoto T Fukuda K : Giant gastric metastasis of malignant melanoma. *Oxf Med Case Rep.* 2021;2021. 10.1093/omcr/omab050 PMC8297646 34306716

[105] Okamoto T Nakano E Yamauchi T : Complete remission in metastatic primary malignant melanoma of the esophagus with nivolumab: a case report. *J Med Case Rep.* 2021;15:345. 10.1186/s13256-021-02928-w 34256852 PMC8278729

[106] Cortellini F Marasco G Renzulli M : Gastric melanoma of unknown primary. *J Gastrointestin Liver Dis.* 2021;30:14. 10.15403/jgld-3420 33723546

[107] Groudan K Ma W Joshi K : Metastatic melanoma presenting as a gastric mass. *Cureus.* 2020;12:e11874. 10.7759/cureus.11874 33415027 PMC7781786

[108] Syed HR Shekar S Aravantagi A : Melanoma and the gastrointestinal (GI) tract: maintaining a high index of suspicion. *Cureus.* 2021;13:e13408. 10.7759/cureus.13408 33628704 PMC7894224

[109] Genova P Sorce M Cabibi D : Gastric and rectal metastases from malignant melanoma presenting with hypochromic anemia and treated with immunotherapy. *Case Rep Oncol Med.* 2017;2017:1–4. 10.1155/2017/2079068 29158932 PMC5660775

[110] Wong K Serafi SW Bhatia AS : Melanoma with gastric metastases. *J Community Hosp Intern Med Perspect.* 2016;6:31972. 10.3402/jchimp.v6.31972 27609722 PMC5016813

[111] Grander LC Cabral F Lisboa AP : Multiple cutaneous melanomas associated with gastric and brain metastases. *An Bras Dermatol.* 2016;91:98–100. 10.1590/abd1806-4841.20164374 28300909 PMC5325008

[112] Carcelain G Rouas-Freiss N Zorn E : In situ T-cell responses in a primary regressive melanoma and subsequent metastases: a comparative analysis. *Int J Cancer.* 1997;72:241–247. 10.1002/(sici)1097-0215(19970717)72:2<241::aid-ijc7>3.0.co;2-r 9219827

[113] Lestre S João A Ponte P : Intraepidermal epidermotropic metastatic melanoma: a clinical and histopathological mimicker of melanoma in situ occurring in multiplicity. *J Cutan Pathol.* 2011;38:514–520. 10.1111/j.1600-0560.2011.01694.x 21352266

[114] Rana SS Chaudhary V Bhasin DK : Narrow band imaging appearance of gastric metastasis from malignant melanoma. *Ann Gastroenterol.* 2013;26. PMC3959478 24714285

[115] Rovere R Souza ME de Hilgert S : Melanoma metastasis to the gastric mucosa preceded by guillain-barré as a paraneoplastic syndrome. *Gastrointest Cancer Res.* 2013;6:150–151. 24312689 PMC3849897

[116] Eivazi-Ziaei J Esmaili H : Metastatic malignant melanoma affecting stomach. *J Cancer Res Ther.* 2014;10:733–736. 10.4103/0973-1482.136029 25313770

[117] El-Sourani N Troja A Raab HR : Gastric metastasis of malignant melanoma: report of a case and review of available literature. *Viszeralmedizin.* 2014;30:273–275. 10.1159/000364814 26288600 PMC4513808

[118] Buissin D Sterle A Schmiegelow P : Primary anorectal malignant melanoma: a rare but aggressive tumor: report of a case. *World J Surg Oncol.* 2015;13:12–014. 10.1186/s12957-014-0419-z 25633933 PMC4326288

[119] Bankar S Patkar S Desai S : Unusual presentation of melanoma of unknown primary origin: a case report and review of literature. *J Cancer Res Ther.* 2015;11. 10.4103/0973-1482.148680 26881591

[120] Krishna Mohan MV Rajappa SJ Reddy TV : Malignant gastrointestinal melanoma with an unknown primary. *Indian J Med Paediatr Oncol.* 2009;30:87–89. 10.4103/0971-5851.60055 20596310 PMC2885878

[121] Farshad S Keeney S Halalau A : A case of gastric metastatic melanoma 15 years after the initial diagnosis of cutaneous melanoma. *Case Rep Gastrointest Med.* 2018;2018. 10.1155/2018/7684964 30151286 PMC6087584

[122] Tapasak B Mcguirt A : Metastatic renal cell carcinoma presenting as chronic bleeding from the stomach: a rare case report. *J Surg Case Rep.* 2022;2022:045. 10.1093/jscr/rjac045 35211289 PMC8863397

[123] Podzolkov VI Pokrovskaya AE Tarzimanova AI : Metastasis of testicular choriocarcinoma in the stomach, complicated by the development of choriocarcinoma syndrome. *Case Rep Gastroenterol.* 2021;15:954–959. 10.1159/000519814 35082590 PMC8740107

[124] Koterazawa S Watanabe J Uemura Y : Solitary synchronous gastric metastasis of renal cell carcinoma. *IJU Case Rep.* 2020;4:53–55. 10.1002/iju5.12239 33426499 PMC7784763

[125] Hakim C Mendelson A Patel J : Metastatic renal cell carcinoma presenting as gastrointestinal bleeding. *Case Rep Gastroenterol.* 2021;15:478–481. 10.1159/000514376 34616243 PMC8454225

[126] Yoshida R Yoshizako T Ando S : Dynamic CT findings of a polypoid gastric metastasis of clear renal cell carcinoma: a case report with literature review. *Radiol Case Rep.* 2020;15:237–240. 10.1016/j.radcr.2019.12.002 31908709 PMC6940632

[127] Bernshteyn M Masood U Smith-Hannah A : Renal cell carcinoma with metastases to the rectum and gastric body. *Proc (Baylor Univ Med Cent).* 2019;33:57–58. 10.1080/08998280.2019.1694389 32063771 PMC6988636

[128] Weissman S Mehta TI Zhornitskiy A : “Homomorphic” tumor metastases as an endodiagnostic clue: a case series of renal-cell carcinoma metastatic to the stomach. *Gastrointest Tumors.* 2019;6:147–152. 10.1159/000502520 31768359 PMC6873029

[129] Chaar A Mouabbi JA Alrajjal A : Metastatic testicular choriocarcinoma: an unusual cause of upper gastrointestinal bleed. *Cureus.* 2019;11:e5243. 10.7759/cureus.5243 31565641 PMC6759043

[130] Arakawa N Irisawa A Shibukawa G : Simultaneous gastric metastasis from renal cell carcinoma: a case report and literature review. *Clin Med Insights Case Rep.* 2018;11. 10.1177/1179547618775095 29844708 PMC5967158

[131] Uehara S Yuasa T Fujisaki J : A case of gastric metastasis from renal cell cancer during the sequential targeted therapy. *Int Cancer Conf J.* 2017;6:114–117. 10.1007/s13691-017-0286-x 31149483 PMC6498289

[132] O’Reilly MK Sugrue G Han-Suyin K : Radiological, pathological and gross correlation of an isolated renal cell carcinoma metastasis to the stomach. *BMJ Case Rep.* 2017;2017. 10.1136/bcr-2017-220469 28478393 PMC5623286

[133] Abu Ghanimeh M Qasrawi A Abughanimeh O : Gastric metastasis from renal cell carcinoma, clear cell type, presenting with gastrointestinal bleeding. *Case Rep Gastrointest Med.* 2017;2017:1–6. 10.1155/2017/5879374 PMC5603082 28951791

[134] Mazumdar S Sundaram S Patil P : A rare case of metastatic germ cell tumor to stomach and duodenum masquerading as signet ring cell adenocarcinoma. *Ann Transl Med.* 2016;4:309. 10.21037/atm.2016.08.20 27668229 PMC5009030

[135] Barras JP Baer H Stenzl A : Isolated late metastasis of a renal cell cancer treated by radical distal pancreatectomy. *HPB Surg.* 1996;10:51–54. 10.1155/1996/56065 9187553 PMC2423831

[136] Riviello C Tanini I Cipriani G : Unusual gastric and pancreatic metastatic renal cell carcinoma presentation 10 years after surgery and immunotherapy: a case report and a review of literature. *World J Gastroenterol.* 2006;12:5234–5236. 10.3748/wjg.v12.i32.5234 16937540 PMC4088027

[137] Hong WS Chung DJ Lee JM : Metastatic gastric linitis plastica from bladder cancer mimicking a primary gastric carcinoma: a case report. *Korean J Radiol.* 2009;10:645–648. 10.3348/kjr.2009.10.6.645 19885323 PMC2770832

[138] Onitilo AA Engel JM Resnick JM : Prostate carcinoma metastatic to the stomach: report of two cases and review of the literature. *Clin Med Res.* 2010;8:18–21. 10.3121/cmr.2010.855 20305145 PMC2842343

[139] Tiwari P Tiwari A Vijay M : Upper gastro-intestinal bleeding - rare presentation of renal cell carcinoma. *Urol Ann.* 2010;2:127–129. 10.4103/0974-7796.68864 20981203 PMC2955230

[140] Yodonawa S Ogawa I Yoshida S : Gastric metastasis from a primary renal leiomyosarcoma. *Case Rep Gastroenterol.* 2012;6:314–318. 10.1159/000338837 22754492 PMC3376334

[141] Chibbar R Bacani J Zepeda-Gómez S : Endoscopic mucosal resection of a large gastric metastasis from renal cell carcinoma. *ACG Case Rep J.* 2013;1:10–12. 10.14309/crj.2013.6 26157808 PMC4435268

[142] Sakurai K Muguruma K Yamazoe S : Gastric metastasis from renal cell carcinoma with gastrointestinal bleeding: a case report and review of the literature. *Int Surg.* 2014;99:86–90. 10.9738/INTSURG-D-13-00115.1 24444276 PMC3897349

[143] Patel H Kumar A Shaaban H : Synchronous metastasis of prostate adenocarcinoma to the stomach and colon: a case report. *N Am J Med Sci.* 2014;6:152–154. 10.4103/1947-2714.128478 24741555 PMC3978939

[144] Sharifi Doloui D Fakharian T Yahyavi V : Primitive neuroectodermal tumor with kidney involvement: a case report. *Iran J Radiol.* 2014;11:e4661. 10.5812/iranjradiol.4661 25035703 PMC4090644

[145] Greenwald D Aljahdli E Nepomnayshy D : Synchronous gastric metastasis of renal cell carcinoma with absence of gastrointestinal symptoms. *ACG Case Rep J.* 2014;1:196–198. 10.14309/crj.2014.50 26157874 PMC4435324

[146] Costa TN Takeda FR Ribeiro U Jr : Palliative laparoscopic resection of renal cell carcinoma metastatic to the stomach: report of a case. *World J Surg Oncol.* 2014;12. 10.1186/1477-7819-12-394 25539876 PMC4364342

[147] Soe AM Bordia S Xiao PQ : A rare presentation of metastasis of prostate adenocarcinoma to the stomach and rectum. *J Gastric Cancer.* 2014;14:271–274. 10.5230/jgc.2014.14.4.271 25580360 PMC4286907

[148] Bhandari V Pant S : Carcinoma prostate with gastric metastasis: a rare case report. *J Cancer Res Ther.* 2015;11:659. 10.4103/0973-1482.139389 26458664

[149] Lowe K Paterson J Armstrong S : Metastatic testicular choriocarcinoma: a rare cause of upper gi bleeding. *ACG Case Rep J.* 2015;3:36–38. 10.14309/crj.2015.94 26504875 PMC4612755

[150] Inagaki C Suzuki T Kitagawa Y : A case report of prostate cancer metastasis to the stomach resembling undifferentiated-type early gastric cancer. *BMC Gastroenterol.* 2017;17:10–93. 10.1186/s12876-017-0655-0 28784100 PMC5547505

[151] Tavukcu HH Aytac O Aktepe F : Ductal adenocarcinoma of the prostate with a rare clinical presentation; late gastric metastasis. *Urol Case Rep.* 2016;7:28–30. 10.1016/j.eucr.2016.03.012 27335785 PMC4909602

[152] Kovecsi A Jung I Bara T : First case report of a sporadic adrenocortical carcinoma with gastric metastasis and a synchronous gastrointestinal stromal tumor of the stomach. *Medicine (Baltimore).* 2015;94:e1549. 10.1097/MD.0000000000001549 26376405 PMC4635819

[153] Koti KA Backianathan S Sebastian P : A rare case of gastric metastasis in ewing’s sarcoma of the femur. *Case Rep Oncol Med.* 2019;2019. 10.1155/2019/2870302 31218087 PMC6537017

[154] Dodis LB Bennett MW Carr-Locke DL : Ewing’s sarcoma metastasis to the gastric wall in a 72-year-old patient. *MedGenMed.* 2006;8. 17406148 PMC1781288

[155] Urakawa H Tsukushi S Tsurudome I : Metastasis of osteosarcoma to stomach made clinically evident by hematemesis: a case report. *World J Surg Oncol.* 2013;11. 10.1186/1477-7819-11-48 23442337 PMC3599039

[156] Shibuya T Osada T Kodani T : Gastrointestinal hemorrhage as the first manifestation of metastatic extragonadal choriocarcinoma. *Intern Med.* 2009;48:551–554. 10.2169/internalmedicine.48.1867 19336957

[157] Tarangelo NP Kistler CA Daitch Z : Synchronous gastric and duodenal metastases from head and neck squamous cell carcinoma: a unique presentation of upper gastrointestinal bleeding. *Ann Gastroenterol.* 2018;31:381–383. 10.20524/aog.2018.0235 29720867 PMC5924864

[158] Kamihara Y Murai S Kikuchi S : Tumor-to-tumor metastasis of diffuse large B cell lymphoma to gastric adenocarcinoma via CXCL12 (SDF-1)/CXCR4 axis: a case report. *BMC Gastroenterol.* 2021;21:270. 10.1186/s12876-021-01844-z 34187383 PMC8243885

[159] Zepeda-Gomez S Camacho J Oviedo-Cardenas E : Gastric infiltration of diffuse large B- cell lymphoma: endoscopic diagnosis and improvement of lesions after chemotherapy. *World J Gastroenterol.* 2008;14:4407–4409. 10.3748/wjg.14.4407 18666335 PMC2731198

[160] Teh JL Wong RK Gowans M : Gastric metastases of oral carcinoma resulting from percutaneous endoscopic gastrostomy placement via the introducer technique. *Gastroenterol Rep (Oxf).* 2013;1:211–213. 10.1093/gastro/got027 24759969 PMC3937992

[161] Elkafrawy A Numan L Albawaliz A : A rare case of metastatic merkel cell carcinoma to the stomach and pancreas presenting with upper gastrointestinal bleeding and obstructive jaundice. *ACG Case Rep J.* 2021;8:e00523. 10.14309/crj.0000000000000523 33521158 PMC7843122

[162] Ha JY Park SE Kim HS : A case report of recurrent Merkel cell carcinoma with synchronous metastases to the heart and stomach. *Medicine (Baltimore).* 2018;97:e13032. 10.1097/MD.0000000000013032 30383666 PMC6221562

[163] Idowu MO Contos M Gill S : Merkel cell carcinoma: a report of gastrointestinal metastasis and review of the literature. *Arch Pathol Lab Med.* 2003;127:367–369. 10.5858/2003-127-0367-MCC 12653587

[164] Parikh MP Samo S Ganipisetti V : Gastric metastasis of Merkel cell carcinoma, a rare cause of gastrointestinal bleeding: case report and review of the literature. *J Gastrointest Oncol.* 2014;5:E68–E72. 10.3978/j.issn.2078-6891.2014.029 25083309 PMC4110492

[165] Subramanian S Kumar M Thulkar S : Bowel metastases from primary leiomyosarcoma of the gluteal region. *Singap Med J.* 2008;49:68–70. 18362989

[166] Dent LL Cardona CY Buchholz MC : Soft tissue sarcoma with metastasis to the stomach: a case report. *World J Gastroenterol.* 2010;16:5130–5134. 10.3748/wjg.v16.i40.5130 20976852 PMC2965292

[167] Samuel T Norly S Ros’aini P : Gastric ulcer that turned out to be metastasis of a synovial sarcoma: a case report and literature review. *Med J Malaysia.* 2016;71:363–365. 28087966

[168] Thorburn D Karim SN Soutar DS : Tumour seeding following percutaneous endoscopic gastrostomy placement in head and neck cancer. *Postgrad Med J.* 1997;73:430–432. 10.1136/pgmj.73.861.430 9338033 PMC2431414

[169] Fuladi R Nagarkar R Roy S : Metastasis to stomach in a patient with anaplastic thyroid carcinoma: a clinical challenge. *Am J Case Rep.* 2019;20:134–138. 10.12659/AJCR.913736 30705249 PMC6368130

[170] Ayaz T Sahin SB Sahin OZ : Anaplastic thyroid carcinoma presenting with gastric metastasis: a case report. *Hippokratia.* 2015;19:85–87. 26435656 PMC4574596

[171] Karrasch T Doppl W Roller FC : Unusual gastric mucosal infiltration by a medullary thyroid carcinoma: a case report. *J Med Case Rep.* 2016;10:208–210. 10.1186/s13256-016-0981-9 27461534 PMC4962496

[172] Ibrahimli A : Metastasis to the Stomach: A Systematic Review.[Dataset]. *OSF.* 2023. 10.17605/OSF.IO/Y4QD5 PMC11066534 38706640

