# Supplementary material for: Metastasis to the stomach: a systematic review
Source: F1000Res. 2023 Oct 18;12:1374. [Version 1] doi: 10.12688/f1000research.140758.1 (PMC11066534; doi:10.12688/f1000research.140758.1)
Supplement: Peer review history

# Reviewer report: Reviewer response for version 1

**DOI:** 10.5256/f1000research.154145.r261964

**Published:** 2024-05-02

**Authors:** Fabio Grizzi

**Affiliations:** 1 Department of Immunology and Inflammation, IRCCS Humanitas Research Hospital, Rozzano, Italy; 2 Department of Biomedical Sciences, Humanitas University, Pieve Emanuele, Italy

I found Ibrahimii et al.'s manuscript on systematically reviewing stomach metastasis quite compelling. Their study stands out for its clarity and significance within its research domain. While I have some minor suggestions for the authors, such as delving deeper into the heterogeneity noted across the included studies and exploring its implications for managing gastric metastasis, I also recommend addressing the variations in terminology among studies, particularly in distinguishing between the term “studies” and “case reports”. This attention to detail will enhance the coherence and applicability of their findings, advancing our understanding of the underlying biology of this neoplastic process.

Are the rationale for, and objectives of, the Systematic Review clearly stated?

Yes

Is the statistical analysis and its interpretation appropriate?

Yes

If this is a Living Systematic Review, is the ‘living’ method appropriate and is the search schedule clearly defined and justified? (‘Living Systematic Review’ or a variation of this term should be included in the title.)

Yes

Are sufficient details of the methods and analysis provided to allow replication by others?

Yes

Are the conclusions drawn adequately supported by the results presented in the review?

Yes

Reviewer Expertise:

My primary area of research lies in investigating cancers originating from unrelated histologies as intricate systems. I am particularly driven by the pursuit of novel prognostic, predictive, and therapeutic biomarkers.

I confirm that I have read this submission and believe that I have an appropriate level of expertise to confirm that it is of an acceptable scientific standard.

# Reviewer report: Reviewer response for version 1

**DOI:** 10.5256/f1000research.154145.r232517

**Published:** 2024-01-02

**Authors:** Murat Sarı

**Affiliations:** 1 Marmara University, Istanbul, Turkey

The stomach is a rare organ in terms of metastasis sites. It would be valuable to write a review evaluating this issue. The review is clearly the result of extensive research. Figures and tables are adequate and informative. Therefore, acceptance of this article will provide impact and citations to the journal.

Are the rationale for, and objectives of, the Systematic Review clearly stated?

Yes

Is the statistical analysis and its interpretation appropriate?

Yes

Are sufficient details of the methods and analysis provided to allow replication by others?

Yes

Are the conclusions drawn adequately supported by the results presented in the review?

Yes

Reviewer Expertise:

Medical oncology

I confirm that I have read this submission and believe that I have an appropriate level of expertise to confirm that it is of an acceptable scientific standard.
